# Egfr signaling promotes juvenile hormone biosynthesis in the German cockroach

**DOI:** 10.1186/s12915-022-01484-z

**Published:** 2022-12-13

**Authors:** Zhaoxin Li, Caisheng Zhou, Yumei Chen, Wentao Ma, Yunlong Cheng, Jinxin Chen, Yu Bai, Wei Luo, Na Li, Erxia Du, Sheng Li

**Affiliations:** 1 Guangdong Provincial Key Laboratory of Insect Developmental Biology and Applied Technology, Institute of Insect Science and Technology & School of Life Sciences, South China Normal University, Guangzhou, China; 2 Guangdong Laboratory for Lingnan Modern Agriculture, Guangzhou, China; 3 Guangmeiyuan R&D Center, Guangdong Provincial Key Laboratory of Insect Developmental Biology and Applied Technology, South China Normal University, Meizhou, China

**Keywords:** Egfr signaling, Pnt, JH, JHAMT, Transcriptional regulation

## Abstract

**Background:**

In insects, an interplay between the activities of distinct hormones, such as juvenile hormone (JH) and 20-hydroxyecdysone (20E), regulates the progression through numerous life history hallmarks. As a crucial endocrine factor, JH is mainly synthesized in the corpora allata (CA) to regulate multiple physiological and developmental processes, including molting, metamorphosis, and reproduction. During the last century, significant progress has been achieved in elucidating the JH signal transduction pathway, while less progress has been made in dissecting the regulatory mechanism of JH biosynthesis. Previous work has shown that receptor tyrosine kinase (RTK) signaling regulates hormone biosynthesis in both insects and mammals. Here, we performed a systematic RNA interference (RNAi) screening to identify RTKs involved in regulating JH biosynthesis in the CA of adult *Blattella germanica* females.

**Results:**

We found that the epidermal growth factor receptor (Egfr) is required for promoting JH biosynthesis in the CA of adult females. The Egf ligands Vein and Spitz activate Egfr, followed by Ras/Raf/ERK signaling, and finally activation of the downstream transcription factor Pointed (Pnt). Importantly, Pnt induces the transcriptional expression of two key enzyme-encoding genes in the JH biosynthesis pathway: juvenile hormone acid methyltransferase (JHAMT) and methyl farnesoate epoxidase (CYP15A1). Dual-luciferase reporter assay shows that Pnt is able to activate a promoter region of *Jhamt*. In addition, electrophoretic mobility shift assay confirms that Pnt directly binds to the − 941~ − 886 nt region of the *Jhamt* promoter.

**Conclusions:**

This study reveals the detailed molecular mechanism of Egfr signaling in promoting JH biosynthesis in the German cockroach, shedding light on the intricate regulation of JH biosynthesis during insect development.

**Supplementary Information:**

The online version contains supplementary material available at 10.1186/s12915-022-01484-z.

## Background

Juvenile hormone (JH), a class of sesquiterpenoid hormones, is primarily synthesized in the corpora allata (CA), a pair of endocrine glands just posterior to the brain of insects. As a crucial endocrine factor, JH regulates insect molting, metamorphosis, reproduction, and many other physiological behaviors [1–5]. During the last century, notable advances have been made in the research of JH signal transduction pathway. In the juvenile stages, JH binds to the JH intracellular receptor methoprene tolerant (Met) and induces the expression of *Krüppel homolog 1* (*Kr-h1*) [6, 7]. Acting as the anti-metamorphic factor, Kr-h1 represses the expression of multiple 20-hydroxyecdysone (20E) primary-response genes (i.e., *Br-C*, *E74*, *E75*, and *E93*), thus preventing 20E-induced premature metamorphosis and maintains the juvenile status [8–13]. Insect female reproduction is mainly governed by JH and 20E [2, 14–16]. Oogenesis is the hallmark of insect female reproduction, consisting of three processes: previtellogenesis, vitellogenesis, and choriogenesis [17]. For most, but not all, insects, JH acts as a gonadotropic hormone to stimulate vitellogenin (Vg) production in the fat body via the JH intracellular signaling as well as yolk protein uptake by developing oocytes via an unidentified membrane receptor [18–22]. Particularly, in hemimetabolous insects, such as the migratory locust, *Locusta migratoria*, the American cockroach, *Periplaneta Americana*, and *B. germanica*, independent of 20E, JH as the main gonadotrophic hormone stimulates vitellogenesis via *Kr-h1* and the polyploidy genes and Vg uptake via the PLC-PKC-ι phosphorylation cascade [18, 23–29]. Because of the important roles of JH in multiple physiological events, JH biosynthesis in insects must be precisely regulated in different developmental stages.

In insects, eight forms of JH have been identified: JH 0, JH I, JH II, JH III, JHB3, JHSB3, 4-methyl-JH I, and methyl farnesoate (MF) [1, 30–32]. Among them, JH III is found in the majority of insects. JH III biosynthesis involves 13 independent enzymes and is conventionally divided into the early mevalonate pathway and late JH-branch steps [33]. Juvenile hormone acid methyltransferase (JHAMT) and methyl farnesoate epoxidase (CYP15A1) are two key regulatory enzymes in the final steps of JH biosynthesis, converting farnesoic acid into JH III [33–35]. Essentially, JH biosynthesis in the CA is considered to be delicately regulated by the expression levels of *Jhamt* and *Cyp15A1*. A series of studies have demonstrated that several neuropeptides (i.e., allatostatins, allatotropins, and short neuropeptide F) and neurotransmitters (i.e., glutamate) are widely involved in regulating JH biosynthesis [36–39]. Transforming growth factor-β signaling and insulin/TOR signaling stimulates JH biosynthesis by upregulating *Jhamt* expression [18, 40, 41], while 20E antagonizes JH signaling to determine developmental transitions by inhibiting *Jhamt* expression [8]. In addition, the transcription factors Mad (Mothers against Dpp), Sex combs reduced (Scr), and POU factor Ventral Veins lacking (Vvl) also stimulate JH biosynthesis during juvenile stages by activating *Jhamt* expression [42–45]. Although the field of JH biosynthesis regulation has made some progress to some extent, its transcriptional regulatory mechanisms remain unclear, especially in the female reproductive stage.

Receptor tyrosine kinases (RTKs) are a large class of enzyme-linked receptors that are expressed on the cell membrane, where they sense extracellular signals and activate a series of biochemical reactions through cascade amplification of intracellular signals [46]. Studies have shown that some RTKs participate in regulating hormone biosynthesis in both insects and mammals. For example, in the fruit fly, *Drosophila melanogaster*, the epidermal growth factor receptor (Egfr) and Torso induce ecdysone (the direct precursor of 20E) biosynthesis by activating the Ras/Raf/MAPK pathway in the prothoracic glands (PG) [47, 48]. Vascular endothelial growth factor receptor-related upregulates the expression of enzyme-encoding genes in the ecdysone biosynthesis pathway to control pupariation timing and body size [49]. In the mouse, fibroblast growth factor 9 activates AKT (protein kinase B, PKB) and MAPK pathways to stimulate the testosterone production of Leydig cells [50]. Recently, we have shown that the insulin receptor (InR) is required for promoting vitellogenesis and oocyte maturation mainly by inducing JH biosynthesis in adult females of *P*. *americana* [25]. Via systematic RNAi screening of RTKs, we here reveal that in addition to *InR*, *Egfr* is involved in promoting the transcriptional expression of *Jhamt* and *Cyp15A1* in adult females of *B. germanica*. On this basis, we further elucidate the detailed molecular mechanism of how Egfr signaling promotes JH biosynthesis in this insect species.

## Results

### RNAi screening of RTKs involved in regulating JH biosynthesis

**Fig. 1:**
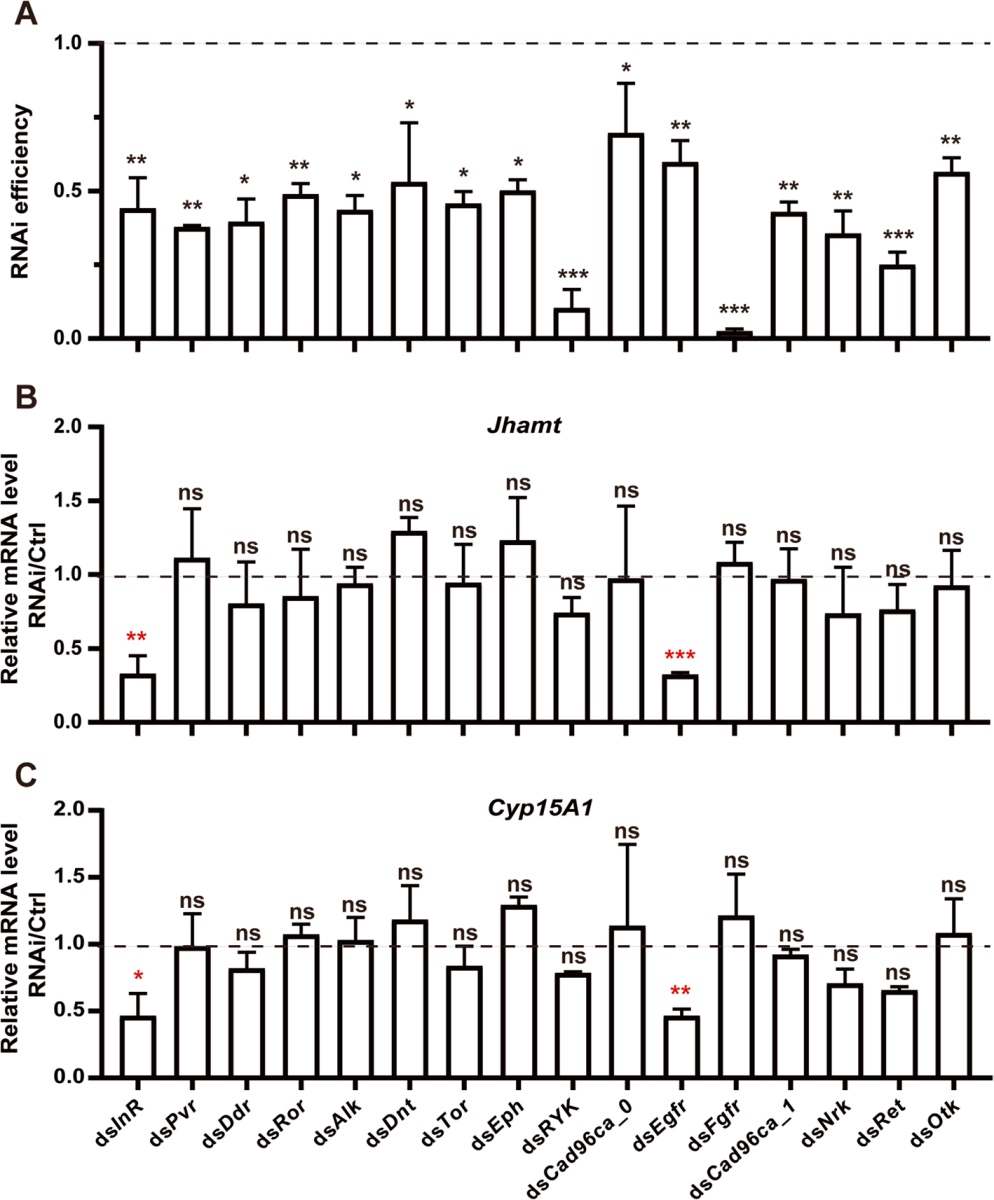
RNAi screening of RTKs involved in regulating JH biosynthesis. **A** qPCR showing the RNAi efficiency of each RTK-encoding gene in the heads of adult females. Injection of dsRNA on days1 and 3 after eclosion; detection on day 5. **B**–**C** qPCR showing the corresponding RNAi effects on the expression levels of *Jhamt* in the heads (**B**) and *Cyp15A1* (**C**). Data are mean ± SD, and *P < 0.05, **P < 0.01, ***P < 0.001, ns (not significant, *P* > 0.05), *n* = 3. *InR*, insulin-like receptor; *Pvr*, PDGF- and VEGF-receptor related; *Ddr*, discoidin domain receptor; *Ror*, RTK-like orphan receptor; *Alk*, anaplastic lymphoma kinase; *Dnt*, Doughnut on 2; *Tor*, Torso; *Eph*, erythropoietin-producing human hepatocellular carcinoma cell line; *RYK*, related-to-tyrosine-kinase; *Cad96Ca-0* and *Cad96Ca-1*, cadherin 96Ca; *Egfr*, epidermal growth factor receptor; *Fgfr*, fibroblast growth factor receptor; *Nrk*, neurotrophic receptor kinase; *Ret*, Ret proto-oncogene; *Otk*, Off-track

JH biosynthesis peaks in the middle-late stage of reproductive cycle in adult females, playing a crucial role in stimulating vitellogenesis and oocyte maturation in *B. germanica* [26, 51–53]. JHAMT and CYP15A1 are the two key regulatory enzymes that catalyze the final two steps of JH biosynthesis, converting farnesoic acid into MF and MF into JH III in this insect species, respectively [33–35]. To explore which RTKs may be involved in regulating JH biosynthesis in the CA of adult females in *B. germanica*, a systematic RNA interference (RNAi) screening against RTKs was performed. A total of 16 RTKs were identified in the German cockroach genome [54]. By injecting with the corresponding dsRNA on days 1 and 3 after eclosion, the transcriptional level of each RTK-encoding gene in the head was downregulated on day 5 (Fig. 1A). Importantly, the transcriptional levels of *Jhamt* and *Cyp15A1* were significantly downregulated only when *InR* or *Egfr* was knocked down, compared with the control (ds*CK*) (Fig. 1B and C). These results show that in addition to InR, Egfr is likely another critical RTK involved in regulating JH biosynthesis in the CA of adult females.

### Egfr is required for promoting JH biosynthesis

**Fig. 2:**
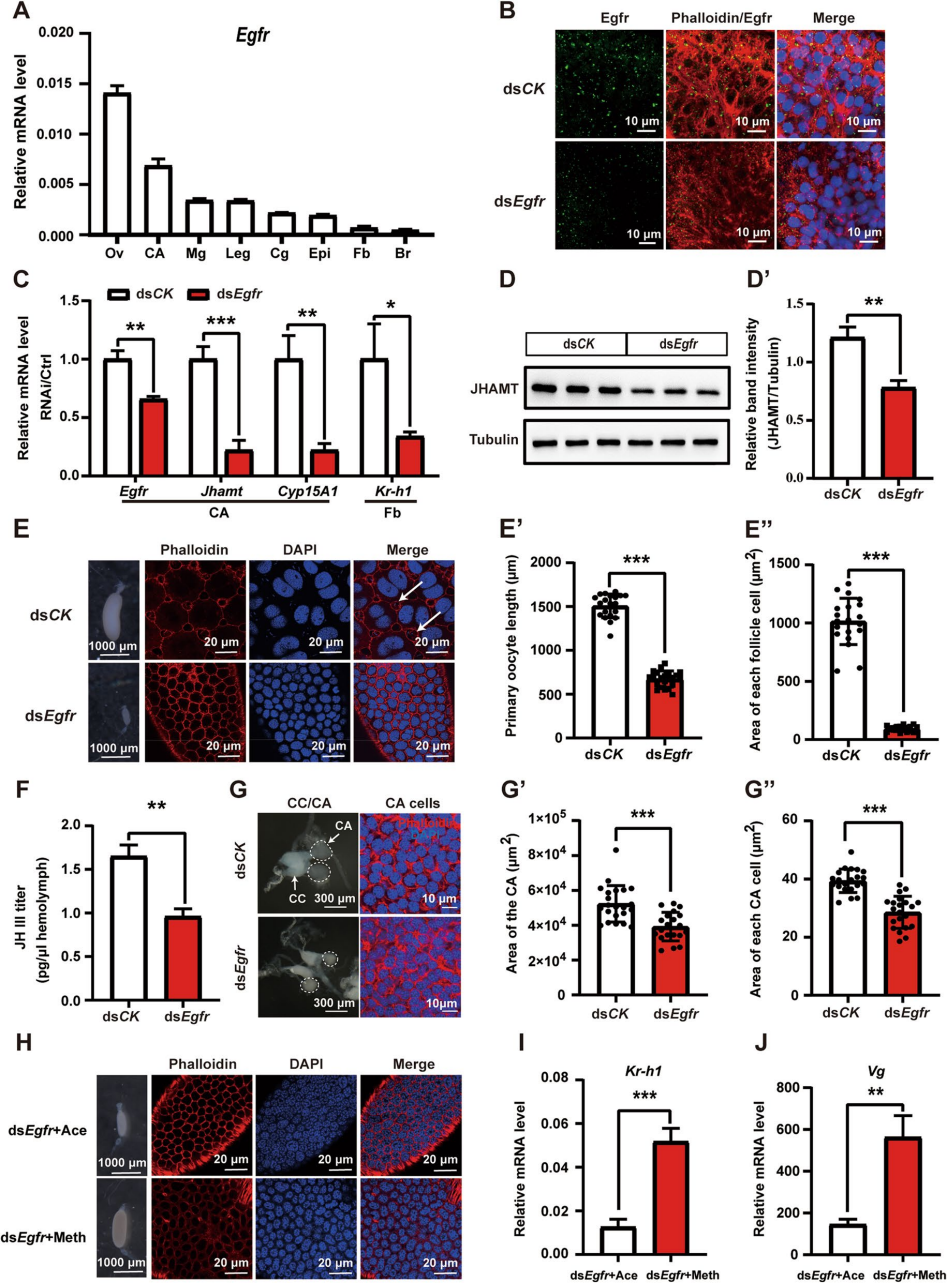
*Egfr* RNAi impairs JH biosynthesis. **A** qPCR showing the tissue-specific expression of *Egfr* on 5-day-old adult females. Ov, ovary; CA, corpora allata; Mg, midgut; Cg, colleterial glands; Epi, epidermis; Fb, fat body; Br, brain. **B** Immunostaining of Egfr protein in the CA cells. Green, Egfr; blue, DNA; red, F-actin; scale bar: 10 μm. **C** qPCR showing the effect of *Egfr* knockdown on the expression levels of *Jhamt* and *Cyp15A1* in the CA and *Kr-h1* in the fat body. **D**–**D’** JHAMT protein levels in the head after *Egfr* RNAi (**D**). Quantification of band intensity of JHAMT protein levels (**D’**). *P < 0.05, **P < 0.01, ***P < 0.001, *n* = 3. **E**–**E”** Effects of *Egfr* RNAi on the size of maturing oocytes and each follicle cell as well as follicular patency formation (**E**). Quantification of the length of maturing oocyte (**E’**) and the area of each follicle cell (**E”**). ***P < 0.001, *n* = 20 or 21. Arrow: follicular patency, blue, DNA; red, F-actin; scale bar: 1000 μm or 20 μm. **F** JH III titer measurements in the hemolymph. **P < 0.01, *n* = 3. **G**–**G** Effects of *Egfr* RNAi on the morphology of CA and size of CA cell (**G**). Blue, DNA; red, F-actin; scale bar: 300 μm or 10 μm. Quantification of the areas of CA (**G’**) and each CA cell (**G”**). ***P < 0.001, *n* = 20 or 21. **H** Effects of treatment with methoprene on the size of ovaries and follicle cells after RNAi *Egfr*. Blue, DNA; red, F-actin; scale bar: 1000 μm or 20 μm. **I**–**J** Effects of treatment with methoprene on the expression of *Kr-h1* and *Vg* in the fat body after RNAi *Egfr*. **P < 0.01, ***P < 0.001, *n* = 3. Ace, acetone; Meth, methoprene

To examine the above hypothesis, we first performed the tissue-specific expression profiling pattern of *Egfr* using real-time quantitative PCR (qPCR). The *Egfr* transcript was expressed in the ovary (Ov), leg, CA, midgut (Mg), epidermis (Epi), fat body (Fb), colleterial gland (Cg), and brain (Br) of adult females, with the most abundant levels in the ovary and CA (Fig. 2A). To further confirm the expression of *Egfr* in the CA, we detected Egfr on day 5 after eclosion using immunostaining with an Egfr antibody. Egfr mainly located on the cell membrane, and a dramatic reduction was observed after injection of *Egfr* dsRNA twice (Fig. 2B). With an approximately 30% reduction in *Egfr* expression on day 5 in the CA, the mRNA levels of *Jhamt* and *Cyp15A1* in the CA were remarkably downregulated (Fig. 2C). *Egfr* downregulation also resulted in a significant downregulation of JHAMT protein expression in the head (Fig. 2D–D’). *Kr-h1* expression levels in the fat body represent JH intracellular signaling, while sizes of the follicle cells and follicular patency represent JH membrane signaling [1, 21, 22, 27, 55]. Consistently, *Egfr* downregulation reduced not only *Kr-h1* expression levels in the fat body (Fig. 2C) but also sizes of the follicle cells and follicular patency in the maturing oocytes (Fig. 2E–E”), indicating the decreases of both JH intracellular and membrane signals. To confirm the above results, JH III titer in the hemolymph, representing JH biosynthesis and secretion, was quantified on day 7 after eclosion using liquid chromatography-mass spectrometry (LC-MS). Likewise, *Egfr* RNAi resulted in a significant decrease of JH III titer (Fig. 2F). Since Egfr signaling is widely involved in cell proliferation and survival, we wondered whether reduced *Egfr* expression impaired CA cell growth and development. Importantly, *Egfr* RNAi led to reduction of the CA size to a certain extent (~20%), and the cell size of the CA declined as well (~25%) (Fig. 2G–G”). We next confirmed whether the phenotype of *Egfr* RNAi could be rescued by JH. Treatment of adult females with JH III analog methoprene caused the size of ovaries and follicle cells to be rescued to some extent, and significantly upregulated the mRNA levels of *Kr-h1* and *Vg* in the fat body (Fig. 2H–J). These data demonstrate that *Egfr* plays a crucial role in promoting JH biosynthesis in the CA, and consequently, JH signals and JH functions.

### Egf ligands vein and Spitz promote JH biosynthesis

**Fig. 3:**
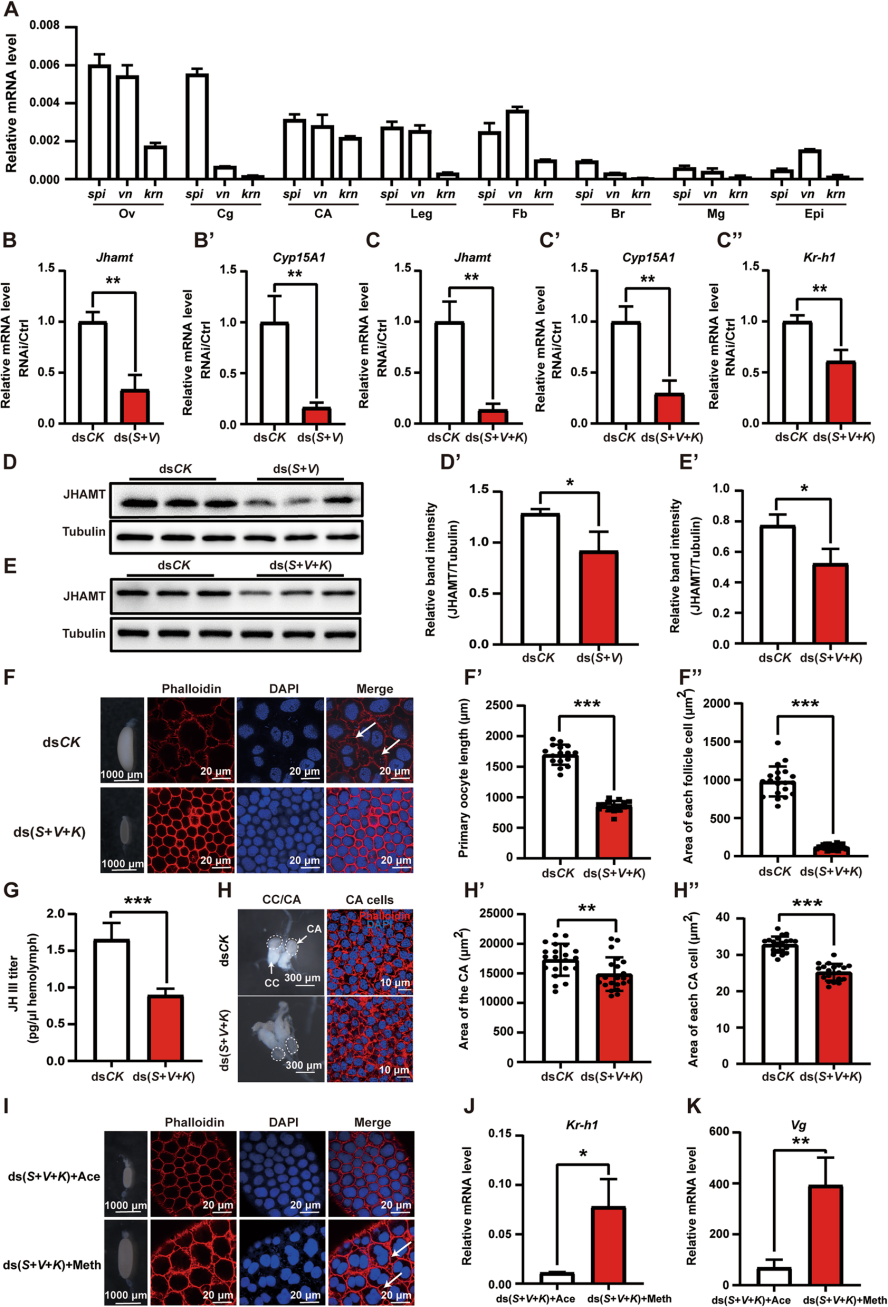
Egf ligands Vn and Spi promote JH biosynthesis. **A** qPCR showing the tissue-specific expression of *spi*, *vn*, and *krn* on 5-day-old adult females. Ov, ovary; CA, corpora allata; Mg, midgut; Cg, colleterial glands; Epi, epidermis; Fb, fat body; Br, brain. **B**–**B’** Effects of simultaneous RNAi *spi* and *vn* on the expression levels of *Jhamt* (**B**) and *Cyp15A1* (**B’**) in the head. **C**–**C”** Effects of simultaneous RNAi *spi*, *vn*, and *krn* on the expression levels of *Jhamt* (**C**) and *Cyp15A1* (**C’**) in the head as well as *Kr-h1* in the fat body (**C”**). **D**–**D’** Effect of simultaneous RNAi *spi* and *vn* on the JHAMT protein levels in the head (**D**). Quantification of band intensity of JHAMT protein level (**D’**). **E**–**E’** Effect of simultaneous RNAi *spi*, *vn*, and *krn* on the JHAMT protein levels in the head (**E**). Quantification of band intensity of JHAMT protein level (**E’**). *P < 0.05, **P < 0.01, ***P < 0.001, *n* = 3. **F**–**F”** Effects of simultaneous RNAi *spi*, *vn*, and *krn* on the size of maturing oocytes and each follicle cell as well as follicular patency formation (**F**). Quantification of the length of maturing oocyte (**F’**) and the area of each follicle cell (**F”**). ***P < 0.001, *n* = 16 or 20. Arrow: follicular patency, blue, DNA; red, F-actin; scale bar: 1000 μm or 20 μm. **G** JH III titer measurements in the hemolymph. ***P < 0.001, *n* = 3. **H**–**H”** Effects of simultaneous *spi*, *vn*, and *krn* RNAi on the morphology of the CA and CA cell (**H**). Blue, DNA; red, F-actin. Scale bar: 300 μm or 10 μm. Quantification of the areas of CA (**H’**) and each CA cell (**H”**). **P < 0.01, ***P < 0.001, *n* = 20 or 21. **I** Effects of treatment with methoprene on the size of ovaries and follicle cells after simultaneous RNAi *spi*, *vn*, and *krn*. Blue, DNA; red, F-actin; scale bar: 1000 μm or 20 μm. **J**–**K** Effects of treatment with methoprene on the expression of *Kr-h1* and *Vg* in the fat body after simultaneous RNAi *spi*, *vn* and *krn*. *P < 0.05, **P < 0.01, *n* = 3. Ace, acetone; Meth, methoprene; *S*, *spi*; *V*, *vn*; *K*, *krn*

In *Drosophila*, Egf ligands, including Gurken (Grk), Spitz (Spi), Vein (Vn), and Keren (Krn), are responsible for Egfr signaling activation in most tissues [56]. In addition, Argos (Aos) serves as a negative ligand antagonist by forming a clamp-like structure around Spi that inhibits Egf signal transduction [57]. Three active types of Egf ligands, Spi, Vn, and Krn, homologous to *Drosophila* were identified in the German cockroach genome [54]. To investigate whether Egf ligands can activate Egfr signaling in the CA and promote JH biosynthesis, we determined the tissue-specific expression profiling of Egf ligands using qPCR. Similar to *Egfr*, the ligand genes *spi*, *vn*, and *krn* were expressed in different tissues (Fig. 3A). Interestingly, with RNAi of each Egf ligand gene alone, the transcript levels of *Jhamt* and *Cyp15A1* were not significantly downregulated (Additional file 1: Fig. S1). We speculated that the downregulation of one of the Egf ligand genes may cause other redundant ligands to activate Egfr signaling in the CA. Therefore, we performed RNAi pairwise combinations of the three active type ligand genes (*spi* and *vn*, *spi* and *krn*, *vn*, and *krn*). *Jhamt* and *Cyp15A1* mRNA levels as well as JHAMT protein levels in the head were significantly downregulated only when *spi* and *vn* were simultaneously knocked down, so did when the three ligand genes were knocked down at the same time (Fig. 3B–E’ and Additional file 1: Fig. S2). Similar to *Egfr* RNAi, simultaneous RNAi knockdown of *spi*, *vn*, and *krn* resulted in significant reductions of *Kr-h1* expression in the fat body (Fig. 3C”), sizes of the follicle cells and follicular patency (Fig. 3F–F”), JH III titer in the hemolymph (Fig. 3G), and CA cell growth and development (Fig. 3H–H”). In addition, methoprene significantly rescued the size of the ovaries and follicle cells and upregulated the mRNA levels of *Kr-h1* and *Vg* in the fat body (Fig. 3I–K), confirming that Egfr signaling promotes JH biosynthesis.

### Egf ligands and Egfr activate the Ras/Raf/ERK signaling pathway in the CA

**Fig. 4:**
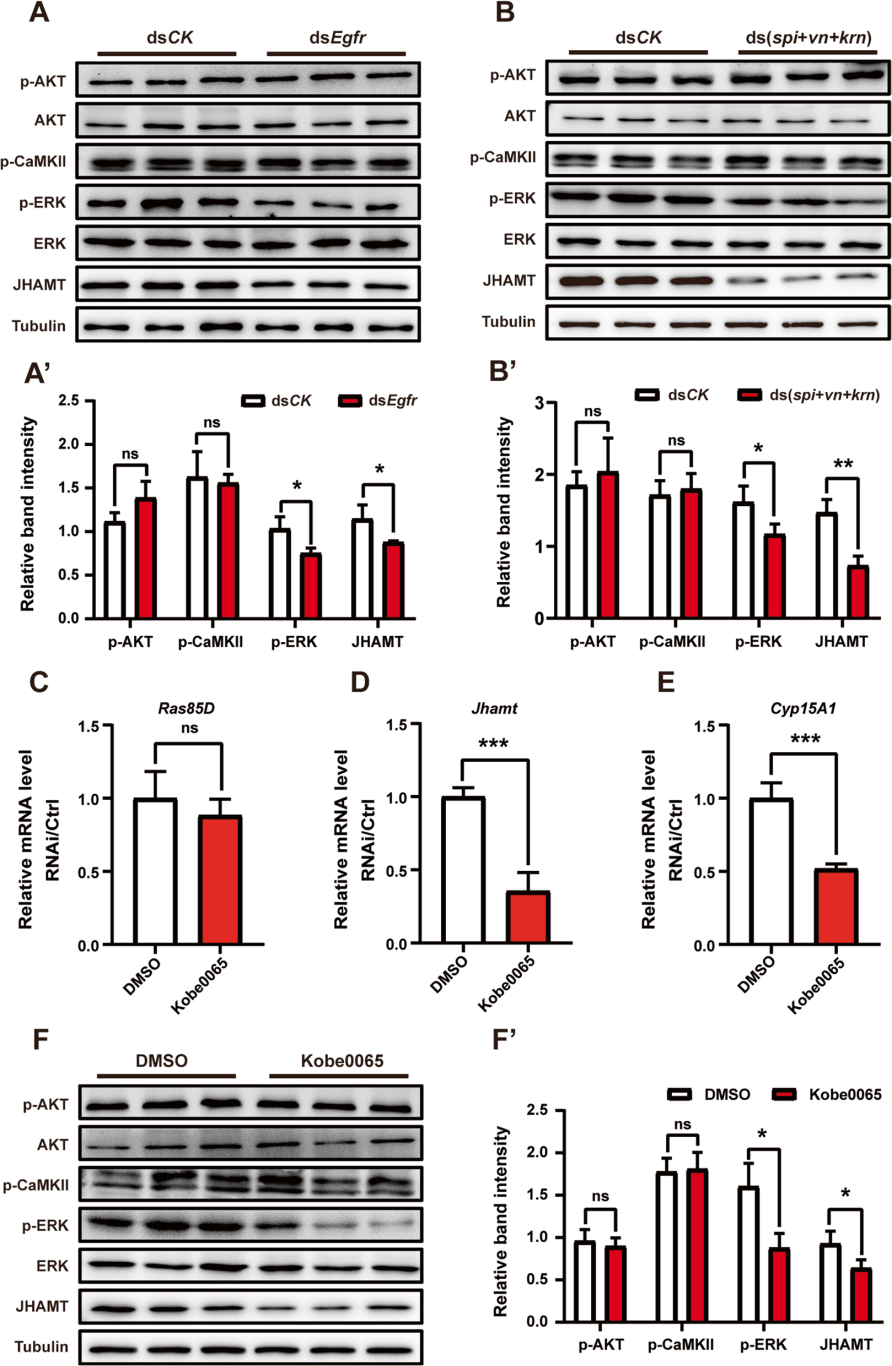
Egf ligands and Egfr activate Ras/Raf/ERK signaling pathway in the CA. **A**–**B”** Western blotting analysis of phospho-AKT, AKT, phospho-CaMKII, phospho-ERK, ERK and JHAMT in the CA after *Egfr* (**A**) or Egf RNAi (**B**). Quantification of the band intensity of phospho-AKT, phospho-CaMKII, phospho-ERK, and JHAMT protein levels (**A’** and **B’**). **C**–**E** Effects of treatment with inhibitor Kobe0065 on the expression levels of *Ras85D* (**C**), *Jhamt* (**D**), and *Cyp15A1* (**E**) in the head. **F**–**F’** Western blotting analysis of phospho-AKT, AKT, phospho-CaMKII, phospho-ERK, ERK, and JHAMT in the CA after treatment with Kobe0065 (**F**). Quantification of the band intensity of phospho-AKT, phospho-CaMKII, phospho-ERK, and JHAMT protein levels (**F’**). *P < 0.05, **P < 0.01, ***P < 0.001, ns (not significant, *P* > 0.05), *n* = 3

There are three main signal transduction pathways activated by Egf ligands, including the PI3K/Akt pathway, PLC pathway, and Ras/Raf/ERK pathway [58, 59]. To explore which pathway was activated by Egf ligands to regulate JH biosynthesis, we detected the phosphorylation levels of AKT, CaMKII, and ERK in the CA, which represent the three signaling pathways, respectively. Our results showed that only p-ERK was significantly reduced when Egf ligand genes or *Egfr* were knocked down (Fig. 4A–B). Therefore, it is likely that Egf ligands may activate the Ras/Raf/MAPK pathway in the CA and thus promote JH biosynthesis. To confirm this hypothesis, we blocked the Ras-Raf interaction by injecting the inhibitor Kobe0065 to interrupt the transduction of Ras/Raf/ERK signaling. Injection with Kobe0065 significantly downregulated the expression of *Jhamt* and *Cyp15A1* but did not affect the expression of *Ras* itself (Fig. 4C–E). Injection with Kobe0065 reduced p-ERK levels in the CA, showing the blockage of Ras-Raf interaction; it also resulted in a significant reduction in JHAMT protein levels (Fig. 4F–F’). The results show that Egf ligands and Egfr mainly activate the Ras/Raf/ERK signaling pathway in the CA and thus promote *Cyp15A1* and *Jhamt* expression and JH biosynthesis.

### Ras/Raf/ERK downstream transcription factor Pnt promotes JH biosynthesis

**Fig. 5:**
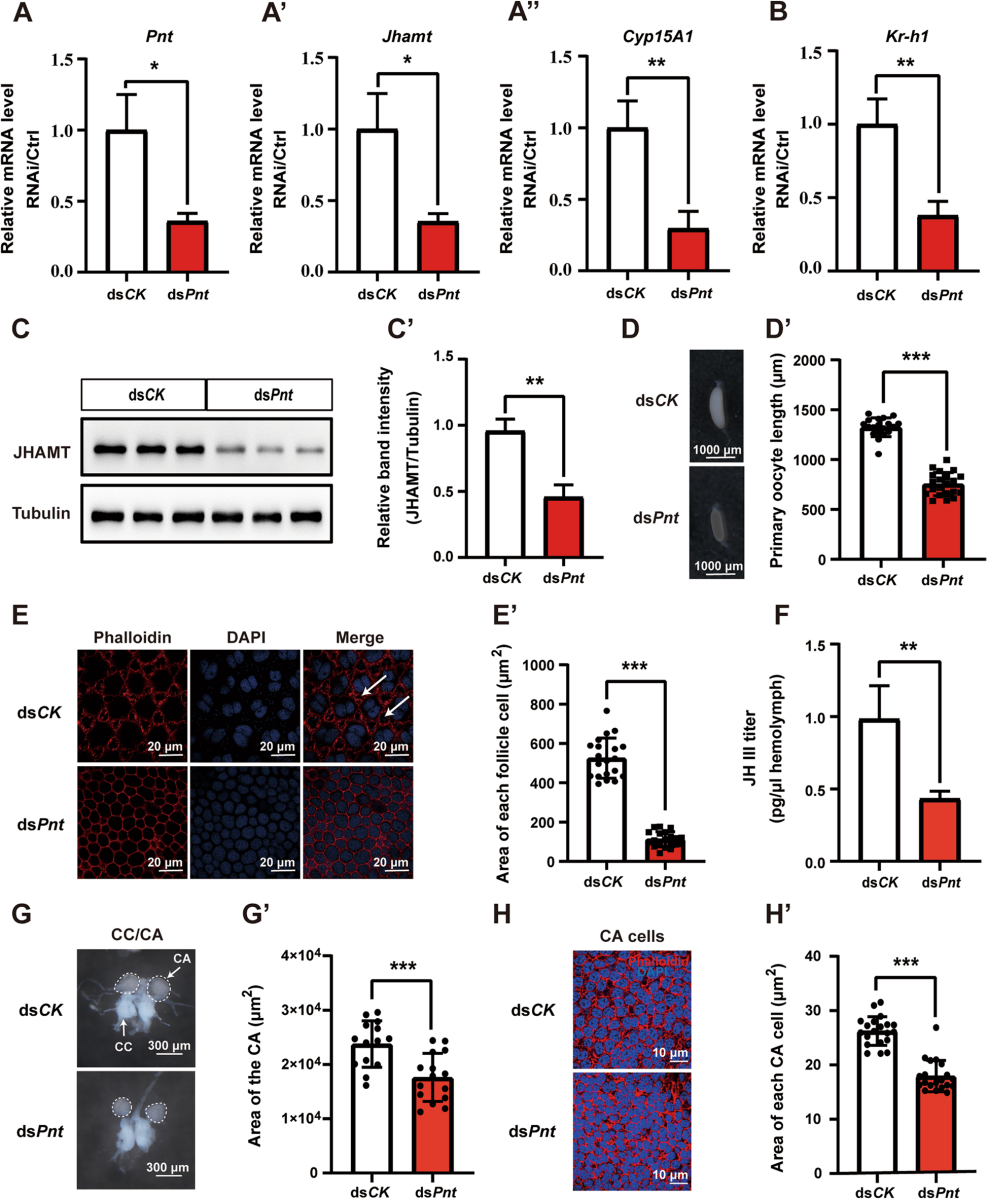
Ras/Raf/ERK downstream transcription factor Pnt promotes JH biosynthesis. **A**–**B** Effects of *Pnt* RNAi on the expression levels of *Jhamt* and *Cyp15A1* in the CA (**A**–**A”**) and *Kr-h1* in the fat body (**B**). **C**–**C’** Western blotting analysis of JHAMT protein level in the head (**C**). Quantification of the band intensity of JHAMT protein levels (**C’**). *P < 0.05, **P < 0.01, *n* = 3. **D**–**E** Effects of *Pnt* RNAi on the size of maturing oocytes (**D**) and each follicle cell as well as follicular patency formation (**E**). Quantification of the length of the maturing oocyte (**D’**) and the area of each follicle cell (**E’**). ***P < 0.001, *n* = 19 or 20. Arrow: follicular patency, blue, DNA; red, F-actin; scale bar: 1000 μm or 20 μm. **F** JH III titer measurements in the hemolymph. **P < 0.01, *n* = 3. **G**–**H** Effects of *Pnt* RNAi on the morphology of CA (**G**) and size of CA cell (**H**). Blue, DNA; red, F-actin; scale bar: 300 μm or 10 μm. Quantification of the area of CA (**G’**) and each CA cell (**H’**). ***P < 0.001, *n* = 15 or 20

Pointed (Pnt) is an ETS-related transcription factor and is a principal nuclear mediator of downstream Ras/Raf/ERK signaling [60]. We then investigated whether Ras/Raf/ERK promotes JH biosynthesis through the downstream transcription factor Pnt. In consistent with the RNAi studies on Egf ligands, Egfr, and the Ras/Raf/ERK signaling, *Pnt* RNAi significantly reduced the expression levels of *Jhamt* and *Cyp15A1* in the CA (Fig. 5A–A”), JHAMT protein levels in the head (Fig. 5C–C’), *Kr-h1* expression levels in the fat body (Fig. 5B), sizes of the follicle cells and follicular patency (Fig. 5D–E), JH III titer in the hemolymph (Fig. 5F), and CA cell growth and development (Fig. 5G–H). The results together suggest that Egf ligands bind Egfr to activate the downstream transcription factor Pnt through Ras/Raf/ERK signaling, eventually inducing *Jhamt* and *Cyp15A1* expression and thus JH biosynthesis.

### Pnt directly binds to Jhamt promoter region and induces Jhamt expression

**Fig. 6:**
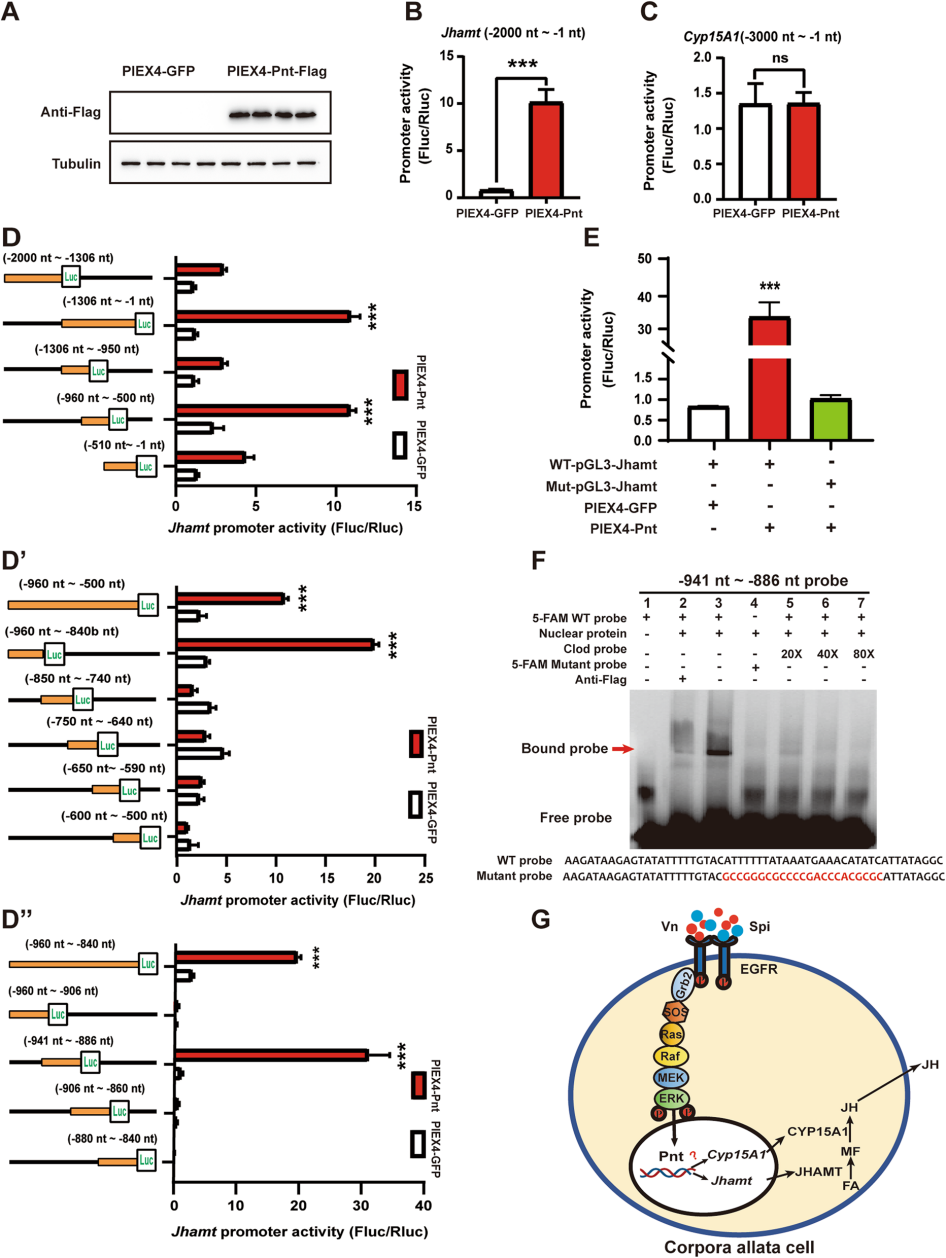
Pnt directly binds to *Jhamt* promoter region and induces *Jhamt* expression. **A** Western blotting confirmed the successful overexpression of Pnt in *Drosophila* KC cells. **B**–**C** Relative luciferase activity of *Jhamt* (− 2000 nt~− 1 nt) (**B**) and *Cyp15A1* (− 3000 nt~− 1 nt) (**C**) promoter transfection with PIEX4-Pnt-Flag. ***P < 0.001, ns (not significant, *P* > 0.05), *n* = 3 or 4. **D**–**D”** Relative luciferase activity of different regions of *Jhamt* promoter. The orange line represents the *Jhamt* promoter fragment that cloned into the pGL3 vector, and the black line represents the *Jhamt* promoter excised fragment. **E** Relative luciferase activity of *Jhamt* promoter mutants. ***P < 0.001, *n* = 3 or 4. **F** EMSA analysis of the binding of nuclear proteins Pnt extracted from KC cell using a region between − 941 and − 886 nt of *Jhamt* promoter and the mutated probes or flag antibody. The mutant nucleotides are marked in red. **G** Schematic representation of the Egf ligands Vn and Spi activate transcription factor Pnt via Ras/Raf/ERK signaling to promote JH biosynthesis in the CA

To investigate whether Pnt directly binds to *Jhamt* and/or *Cyp15A1* promoter regions and thus induces their expression, we performed dual-luciferase reporter assay and electrophoretic mobility shift assay (EMSA) using *Drosophila* KC cells. As detected by western blotting with a Flag antibody, Pnt-Flag was successfully overexpressed in *Drosophila* KC cells (Fig. 6A). Dual-luciferase reporter assay revealed that luciferase activity significantly increased when *Jhamt* (− 2000 nt~− 1 nt)-pGL3 and PIEX4-Pnt-Flag were co-transfected in KC cells but not when *Cyp15A1* (− 3000 nt~− 1 nt)-pGL3 and PIEX4-Pnt-Flag were co-transfected, indicating that Pnt is able to directly bind to the − 2000 nt~− 1 nt promoter region of *Jhamt* (Fig. 6B and C). To identify the key region for Pnt binding, we continuously truncated the *Jhamt* promoter region and finally identified that the *Jhamt* promoter − 941~− 886 nt region significantly enhanced luciferase activity in KC cells (Fig. 6D–D”). Pnt has a conserved ETS DNA binding domain that can specifically bind to purine-rich DNA motifs [61]. Therefore, we mutated the AT-rich region of the − 941~− 886 nt *Jhamt* promoter and found that its promoter activity was significantly reduced (Fig. 6E). To further assess the role of Pnt in inducing *Jhamt* expression, we performed an in vitro EMSA experiment to investigate whether *Jhamt* was a direct target of Pnt. EMSA results showed that the labeled − 941~ − 886 nt probe specifically bound the nuclear proteins extracted from the PIEX4-Pnt-Flag overexpressing cells (Fig. 6F, lane 3). The specific super-shift band did not appear when anti-Flag was added and incubated together (Fig. 6F, lane 2), possibly because anti-Flag blocked the binding site of the probe and Pnt. Moreover, the addition of the mutated probe effectively eliminated the binding of the Pnt-Flag fusion protein to the DNA (Fig. 6F, lane 4), and the addition of excess cold competitive probe effectively weakened the binding band (Fig. 6F, lanes 5–7). These results show that Pnt directly binds to the promoter region of *Jhamt* and thus induces *Jhamt* expression.

## Discussion

### Multiple upstream signals coordinately regulate JH biosynthesis to ensure female reproductive success

JH contents and functions are obviously different in distinct developmental stages. For example, in adult females, reproduction as the first priority is mainly regulated by JH in hemimetabolous insects, including *B. germanica* [15, 62, 63]. During the first reproductive cycle in adult females of this insect species, JH biosynthesis is low at the beginning, gradually rises and peaks at the middle-late stage and rapidly decreases thereafter (Additional file 1: Fig. S3) [51]. To ensure female reproductive success, JH biosynthesis must be coordinately regulated by multiple upstream signals. In this article, we discover that in addition to InR, Egfr is another RTK promoting JH biosynthesis in the CA during the female reproductive stage. Egfr plays an equally important role as InR in JH biosynthesis during the first reproductive cycle of adult females (Additional file 1: Fig. S4), although, throughout the first reproductive cycle of adult females, *Egfr* did not change significantly in the CA. But, the expression patterns of *spi* and *Pnt* were well correlated with the JH level (Additional file 1: Fig. S3). We assume that many more factors (i.e., neuropeptides, neurotransmitters, Dpp and 20E) should participate in the precise regulation of JH biosynthesis in this process. It is necessary to note that Egfr is likely involved in ovarian development in a direct manner or by interacting with other signal pathways [64, 65]. Therefore, in this study, we did not use ovary size as a hallmark for Egfr signaling in the regulation of JH biosynthesis.

### Transcriptional regulation of Jhamt expression and JH biosynthesis

Recently, a series of studies have indicated that the transcriptional regulatory mechanism of JH biosynthesis is likely coordinated by some transcription factors. In both the silkworm, *Bombyx mori*, and the red flour beetle, *Tribolium castaneum*, Vvl regulates the expression of both JH and ecdysone biosynthesis genes [44, 45, 66]; moreover, Vvl directly binds to a cis-regulatory element (CRE) of the *Jhamt* promoter [45]. In *B. mori*, the homeodomain transcription factor Scr binds to a CRE of the *Jhamt* promoter and activates JH biosynthesis [43]. In addition, Dpp also stimulates JH biosynthesis through the transcription factor Mad in *Drosophila* and the cricket, *Gryllus bimaculatus* [40, 67]. Here, in *B. germanica*, we demonstrate that Pnt directly binds to a CRE in the *Jhamt* promoter and thus induces *Jhamt* expression (Fig. 6). However, Pnt did not drive the activation of the *Cyp15A1* promoter in the region of 1-3000 nt (Fig. 6C). Therefore, we speculate that the site of the Pnt binding *Cyp15A1* promoter region is not within this 3000 nt. Another possibility is that Pnt regulation of *Cyp15A1* expression is indirect or through crosslinking with other genes or pathways. By receiving different upstream signals, multiple transcription factors are likely involved in the precise regulation of *Jhamt* expression and JH biosynthesis. The transcriptionally regulatory mechanism might vary in distinct developmental stages and different insect species.

### Egfr signaling has distinct functions in different tissues

In *Drosophila*, Egf ligands, including Grk, Spi, Vn, and Krn, are responsible for Egfr functions in stage- and tissue-specific contexts [56]. For example, Spi and Krn act cooperatively in eye and embryo development and midgut progenitor proliferation [68–70]. Vn is required for global growth of the early *Drosophila* wing disc and the distal leg region [71, 72]. Asymmetric localization of Grk is critical for its function in anterior-posterior and dorsal-ventral polarity in the egg and embryo [73, 74]. It is necessary to note that the absence of one of the Egf ligands may cause other redundant ligands to activate Egfr signaling (Fig. 3, Additional file 1: Fig. S1 and Fig. S2) [68, 75]. In addition to its regulatory functions in cell growth, proliferation, and differentiation, some recent studies have indicated that Egfr signaling promotes ecdysone biosynthesis in the PG of both *Drosophila* and *T. castaneum* [47, 75]. In this study, we not only demonstrate that the Egf ligands Vn and Spi have a redundant function to regulate *Jhamt* and *Cyp15A1* expression and JH biosynthesis in adult females but also show that *Egfr* signaling transcriptionally regulates both JH and 20E biosynthesis at the nymph stages (Additional file 1: Fig. S5). Disruption of Egfr signaling at the nymphal stages resulted in high mortality and serious molting defects. The phenotypic defects of RNAi *Egfr* and Egf ligands in the nymph were similar to *InR* RNAi. Because either *Egfr* or *InR* RNAi affected nymph growth and development by reducing both JH and 20E biosynthesis, neither precocious nor supernumerary nymphs were observed (Additional file 1: Fig. S5 and Fig. S6) [18]. Therefore, Egfr signaling should play a variety of stage- and tissue-specific roles during insect development.

## Conclusions

JH and 20E are two primary endocrine hormones in insects. JH induces of expression of *Kr-h1* that antagonizes 20E signaling to prevents premature metamorphosis and maintain juvenile status, while JH is a gonadotropic hormone of adult females in most, but not all, insects [2, 4, 9, 13]. In the adult female of the German cockroach, we here demonstrate that Egf ligands Vn and Spi activate downstream transcription factor Pnt via Ras/Raf/ERK signaling to induce the transcriptional expression of *Jhamt* and *Cyp15A1*. Moreover, Pnt can directly bind to the promoter region of *Jhamt* and thus promote JH biosynthesis. This study reveals the detailed molecular mechanism of Egfr signaling in promoting JH biosynthesis, shedding light on that JH biosynthesis must be delicately regulated throughout the insect life cycle.

## Methods

### Insects

The line of *B. germanica* was collected from Shanghai as previously described [76]. Cockroaches were kept at 28 °C and at a relative humidity of 60%, with a 12:12 h light/dark photoperiodic regime in a small plastic jar. Cockroaches were fed with commercial rat chow (Keao Xieli, Beijing, China) and tap water. For synchronization of developmental time, cockroaches that emerged within 2 h were collected in a small plastic jar. The day of ecdysis was adopted as day 1.

### Total RNA extraction and qPCR

Total RNA was extracted from the ovary, CA, epidermis, midgut, colleterial glands, fat body, or brain using TRIzol™ Reagent (Invitrogen, MA, USA). Total RNA (2 μg) was reverse transcribed into cDNA using a SMARTer® PCR cDNA Synthesis Kit (Takara, Dalian, China), following the manufacturer’s instructions. qPCR was performed using Hieff® qPCR SYBR Green Master Mix (Low Rox Plus) (Yeasen Biotech, Shanghai, China) and Applied Biosystems™ QuantStudio™ 6 Flex Real-Time PCR System (Thermo Fisher Scientific, MA, USA). The thermocycling conditions were as follows: 94 °C for 2 min, 40 cycles of 94°C for 10 s, and 56 °C for 30 s. Actin-5c was used as an internal reference [76]. The relative expression levels of the indicated genes were computed using the 2-ΔΔCt method [25, 76]. The primer sequences used for qPCR are listed in Table S1 (Additional file 1).

### JH III extraction and quantification

The appendages (three pairs of jointed legs) of adult females on day 5 or day 7 after eclosion were removed, placed in a centrifuge tube (one tube for 30 insects), and centrifuged at 4 °C, 6000 g for 5 min. Approximately 100 μl of hemolymph was collected, the hemolymph was mixed with the same volume of benzonitrile, and added with 100 μl of 0.9% NaCl and 200 μl of N-hexane, and then the mixture was centrifuged at 4 °C and 5000 g for 5 min. The supernatant was dried with nitrogen, and the dried powder was dissolved in 50% methanol. The extracted JH III was quantified using a SCIEX QTRAP 4500 MD (SCIEX, Toronto, Canada) tandem mass spectrometer with a Shimadzu Exion LC UHPLC system. A Waters BEH C18 column (130 Å, 1.7 μm, 2.1 mm X 50 mm column) was used for separation. The mobile phase consisted of solvents A and B (water/acetonitrile/formic acid (A: 98/2/0.1%; B: 2/98/0.1%)), and the elution gradient of phase B rises from 10% to 85% within 10 min. Electrospray positive ion mode was used for mass spectrometry detection, and multiple response monitoring (MRM) scanning mode was selected for targeted quantification of JH III [77].

### RNAi

For in vivo RNAi, different target gene fragments were PCR amplified with 2×Hieff®PCR Master Mix (With Dye) (Yeasen Biotech, Shanghai, China). The corresponding gene fragment was cloned into the pMD™18-T Vector (Takara, Dalian, China) for sequencing to ensure the accuracy of the PCR amplification. The T7 promoter sequence was linked to the 5*′* end of the corresponding dsRNA primer, and the PCR amplified product was used to synthesize the dsRNA template. The dsRNA was synthesized and purified using the T7 RiboMAX Express RNAi kit (Promega, WI, USA) according to the manufacturer’s instructions. Control dsRNA (CK, a 92 bp noncoding sequence from the pSTBlue-1 vector) was used [78, 79]. A volume of 2 μl of dsRNA (2 μg/μl) was injected into the abdomen of 1-day-old adult females and 3-day-old adult females with a 10 μl Hamilton microsyringe [25, 76]. All primers used for dsRNA synthesis in this study are summarized in Table S1 (Additional file 1).

### Kobe0065 and methoprene application

A female adult was injected with 4 μg (2 μg/μl) Kobe0065 (MCE, NJ, USA) on 1-day-old and 3-day-old adult females, respectively. The controls were injected with the corresponding volume of the solvent. In the rescue experiment, at 24 h after *Egfr* RNAi, a total of 40 μg (10 μg/μl) methoprene (MCE, NJ, USA) was applied to the abdomen of the adult females, and acetone was applied to the control. qPCR or Western blot analyses were performed on D5 of adult females.

### Western blotting

Total proteins were extracted from distinct tissues with RIPA Lysis Buffer (Beyotime Biotechnology, China) with 1% (v/v) 1 mM phenylmethylsulfonyl fluoride (PMSF). Extracted proteins were quantified using a Bradford Protein Quantification Kit, (Yeasen Biotech, Shanghai, China). Approximately 10 μg of protein per lane was separated with 10% SDS-PAGE and then transferred to polyvinylidene difluoride membranes (PVDF) (Millipore, MA, USA). The primary antibodies used in this study, including anti-JHAMT, were obtained by immunizing rabbits with purified JHAMT protein (ABclonal, Wuhan, China). In brief, the full-length CDS encoding the JHAMT was cloned into the pET28a vector, expressed in *Escherichia coli* with 6×His tag at the N terminus. The recombinant JHAMT protein was then affinity purified through Ni-chelating chromatography and used as an antigen for antibody production. Because of the identical amino acid sequences, anti-phospho-AKT (Ser473) (Cell Signaling Technology, USA), anti-AKT (Cell Signaling Technology, MA, USA), anti-phospho-ERK (Cell Signaling Technology, MA, USA), anti-ERK (Cell Signaling Technology, MA, USA), anti-phospho-CaMKII (Cell Signaling Technology, MA, USA), and anti-tubulin (Beyotime Biotechnology, Shanghai, China) were used. All primary antibodies were diluted to 1:2000 and incubated overnight at 4 °C. Goat anti-mouse or anti-rabbit lgG-HRP-conjugated (Beyotime Biotechnology, Shanghai, China) was used as a secondary antibody. Protein was finally detected by chemiluminescence using Immobilon western HRP substrate (Millipore, MA, USA). Images were obtained using a Tanon-5500 Chemiluminescent Imaging System (Tanon, Shanghai, China) and quantitatively measured from Western blots using ImageJ [25, 76].

### Immunohistochemistry

Tissues were dissected and fixed in 4% paraformaldehyde at 25 °C for 60 min, washed three times with PBST (PBS containing 0.3% Triton-X 100 (v/v)), and then incubated with anti-Egfr (1: 200, Abcam, Cambridge, UK) at 4 °C overnight. The secondary antibodies used Alexa Fluor 488 goat anti-rabbit IgG (1:400, Invitrogen, MA, USA) at 25 °C for 1 h and washed thrice with PBST. Nuclei and F-actin were stained with DAPI (1:2000, Yeasen Biotech, China) and TRITC Phalloidin (1:2000, Yeasen Biotech, Shanghai, China). Images were obtained with an Olympus Fluoview FV3000 confocal laser scanning microscope (Olympus, Tokyo, Japan) [8, 25].

### 5′-rapid amplification of cDNA ends (5′-RACE)

A of total 1 μg RNA of the female’s head was used for 5*′*-RACE-Ready first-strand cDNA synthesis according to the SMARTer RACE 5*′*/3*′* Kit (Takara, Dalian, China) manufacturer’s instructions. 5*′* RACE was first performed using the in-built universal primer mix (UPM) and a gene-specific primer in Table S1 (Additional file 1) and SeqAmp DNA polymerase [76]. PCR amplification procedures were performed according to the instructions provided by the supplier. The PCR products were gel-purified and cloned into a pTOPO-Blunt Vector (Aidlab, Beijing, China). The complete 5*′* UTR sequence was obtained according to the sequencing results.

### Dual-luciferase reporter assay

PCR was used to amplify the different regions of the *Jhamt* or *Cyp15A1* promoter. These variant fragments were then inserted into a linearized pGL3-basic vector (Promega, WI, USA). *Pnt* was cloned into a PIEX4 expression vector (Invitrogen, MA, USA) for overexpression in *Drosophila* KC cells. The pGL3 reporter vector carrying an indicated promoter region and a reference reporter plasmid of pRL-SV40 were co-transfected with PIEX4-Pnt-Flag or PIEX4-GFP (control) into KC cells and then incubated in 96-well plates at 28 °C for 48 h. Luciferase activity was normalized to *Renilla* luciferase activity and determined using the Dual-Luciferase® Reporter (DLR™) Assay System and a GloMax 96 Microplate Luminometer (Promega, WI, USA) [76].

### EMSA

Pnt protein was overexpressed by the PIEX4-Pnt-Flag vector in KC cells, and nuclear proteins were extracted from the KC cells using NE-PER^TM^ Nuclear and Cytoplasmic Extraction Reagents (Thermo, MA, USA). The − 941~− 886 nt fragment from the *Jhamt* promoter region was labeled with 5-FAM. DNA oligonucleotides were annealed at 60 °C for 30 min to produce double-stranded probes. Double-stranded DNA was used as a probe for EMSA. EMSA was performed using a Light Shift ^TM^ EMSA Optimization & Control Kit (Thermo, MA, USA). DNA-protein binding assays were performed in a 20-μl system containing 8 μl of the reaction mix, 8 μl of nucleoprotein (2μg/μl), 1 μl of the probe (40 μmol), and 2 μl of 10x binding buffer at 25 °C for 40 min. For the competition assay, 20-fold, 40-fold, and 80-fold excess of unlabeled wild-type probe was added to the reaction system mentioned above for 10 min and then added to the labeled probe for 30 min. For the mutant assay, the labeled mutant probe was added to the reaction system for 40 min. In the antibody-based assay, the labeled probe, nucleoprotein, and 1 μl of Flag antibody were incubated at 25 °C for 40 min. The DNA-protein complex was separated on a 5% nondenaturing polyacrylamide gel in 0.5x TBE buffer (Beyotime Biotechnology, Shanghai, China) by electrophoresis at 100 V for 80 min. Finally, images were obtained with a Tanon-5500 Chemiluminescent Imaging System (Tanon, Shanghai, China).

### Data analyses and statistics

Statistical analyses were performed with Student’s t-test using IBM SPSS Statistics 19.0 software. ***P < 0.001; **P < 0.01; *P < 0.05. Data are presented as mean ± SD of at least three independent biological replicates.

## Supplementary Information

**Additional file 1: Figure S1.** Effect of RNAi each Egf ligand gene on *Jhamt* and *Cyp15A1* expression. **Figure S2.** Effect of RNAi pairwise combinations of the three Egf ligand genes *Jhamt* and *Cyp15A1* expression. **Figure S3.** Expression patterns of *Egfr*, *spi*, *vn*, *Jhamt* and *Cyp15A1* of adult females, during the first vitellogenic cycle. **Figure S4.** Compare *Egfr* RNAi with *InR* RNAi. **Figure S5.** RNAi *Egfr* at the nymph stage. **Figure S6.** Egf ligands and *Pnt* RNAi at the nymph stage. **Figure S7.** Specificity verification of anti-JHAMT. **Table S1.** Primers used for qPCR, RNAi and 5*′*-RACE.

**Additional file 2. **The individual data values for Fig. 1A-C, Fig. 2A, C, D’, E’- E”, F, G’-G”, I, J, Fig. 3, A, B-B’, C-C”, D’, E’, F’-F”, G, H’-H”, J, K, Fig. 4A’-B’, C-E, F’, Fig. 5A-A”, B, C’, D’, E’, F, G’, H’, Fig. 6B-C, D-D”, E, Fig. S1A-A”, B-B”, C-C”, D’-F’, Fig. S2A-A’, B-B’, C’-D’, Fig. S3A-D, Fig. S4B-E, Fig. S5B-E and Fig. S6B-C.

**Additional file 3. **Original Western blot data.

## Data Availability

All data generated or analyzed during this study are included in this published article and its supplementary information files. The datasets used and/or analyzed during the current study are available from the corresponding author on reasonable request.

## Abbreviations

JH: Juvenile hormone
20E: 20-Hydroxyecdysone
CA: Corpora allata
Egfr: Epidermal growth factor receptor
RNAi: RNA interference
qPCR: Real-time quantitative PCR
Ov: Ovary
Mg: Midgut
Epi: Epidermis
Fb: Fat body
Cg: Colleterial gland
Br: Brain
Pnt: Pointed
JHAMT: Juvenile hormone acid methyltransferase
CYP15A1: Methyl farnesoate epoxidase
Met: Methoprene tolerant
Kr-h1: Krüppel homolog 1
Vg: Vitellogenin
MF: Methyl farnesoate
Mad: Mothers against Dpp
Scr: Sex combs reduced
Vvl: Ventral Veins lacking
RTKs: Receptor tyrosine kinases
PG: Prothoracic glands
AKT: Protein kinase B
InR: Insulin receptor
LC-MS: Liquid chromatography-mass spectrometry
Grk: Gurken
Spi: Spitz
Vn: Vein
Krn: Keren
Aos: Argos
EMSA: Electrophoretic mobility shift assay
CRE: Cis-regulatory element
Pvr: PDGF- and VEGF-receptor related
Ddr: Discoidin domain receptor
Ror: RTK-like orphan receptor
Alk: Anaplastic lymphoma kinase
Dnt: Doughnut on 2
Tor: Torso
Eph: Erythropoietin-producing human hepatocellular carcinoma cell line
RYK: Related-to-tyrosine-kinase
Cad96Ca: Cadherin 96Ca
Fgfr: Fibroblast growth factor receptor
Nrk: Neurotrophic receptor kinase
Ret: Ret proto-oncogene
Otk: Off-track
