# Supplementary material for: Egfr signaling promotes juvenile hormone biosynthesis in the German cockroach
Source: BMC Biol. 2022 Dec 13;20:278. doi: 10.1186/s12915-022-01484-z (PMC9749228; doi:10.1186/s12915-022-01484-z)
Supplement: Supplementary file 1 — Additional file 1: Figure S1. Effect of RNAi each Egf ligand gene on Jhamt and Cyp15A1 expression. Figure S2. Effect of RNAi pairwise combinations of the three Egf ligand genes Jhamt and Cyp15A1 expression. Figure S3. Expression patterns of Egfr, spi, vn, Jhamt and Cyp15A1 of adult females, during the first vitellogenic cycle. Figure S4. Compare Egfr RNAi with InR RNAi. Figure S5. RNAi Egfr at the nymph stage. Figure S6. Egf ligands and Pnt RNAi at the nymph stage. Figure S7. Specificity verification of anti-JHAMT. Table S1. Primers used for qPCR, RNAi and 5′-RACE. [file 12915_2022_1484_MOESM1_ESM.docx]

**Egfr signaling** **promotes juvenile hormone biosynthesis in the German cockroach**

Zhaoxin Li^1,2,3,#^, Caisheng Zhou^1,#^, Yumei Chen^1^, Wentao Ma^1^, Yunlong Cheng^1^, Jinxin Chen^1^, Yu bai, Wei Luo^1^, Na Li^1,3,*^, Erxia Du^1,2,*^, and Sheng Li^1,2,3,*^

^1^Guangdong Provincial Key Laboratory of Insect Developmental Biology and Applied Technology, Institute of Insect Science and Technology & School of Life Sciences, South China Normal University, Guangzhou, China

^2^Guangdong Laboratory for Lingnan Modern Agriculture, Guangzhou, China

^3^Guangmeiyuan R&D Center, Guangdong Provincial Key Laboratory of Insect Developmental Biology and Applied Technology, South China Normal University, Meizhou, China

^#^ These authors contributed equally

^*^Authors for correspondence: lina5hs@m.scnu.edu.cn, duerxia@m.scnu.edu.cn, and [lisheng@scnu.edu.cn](mailto:lisheng@scnu.edu.cn)


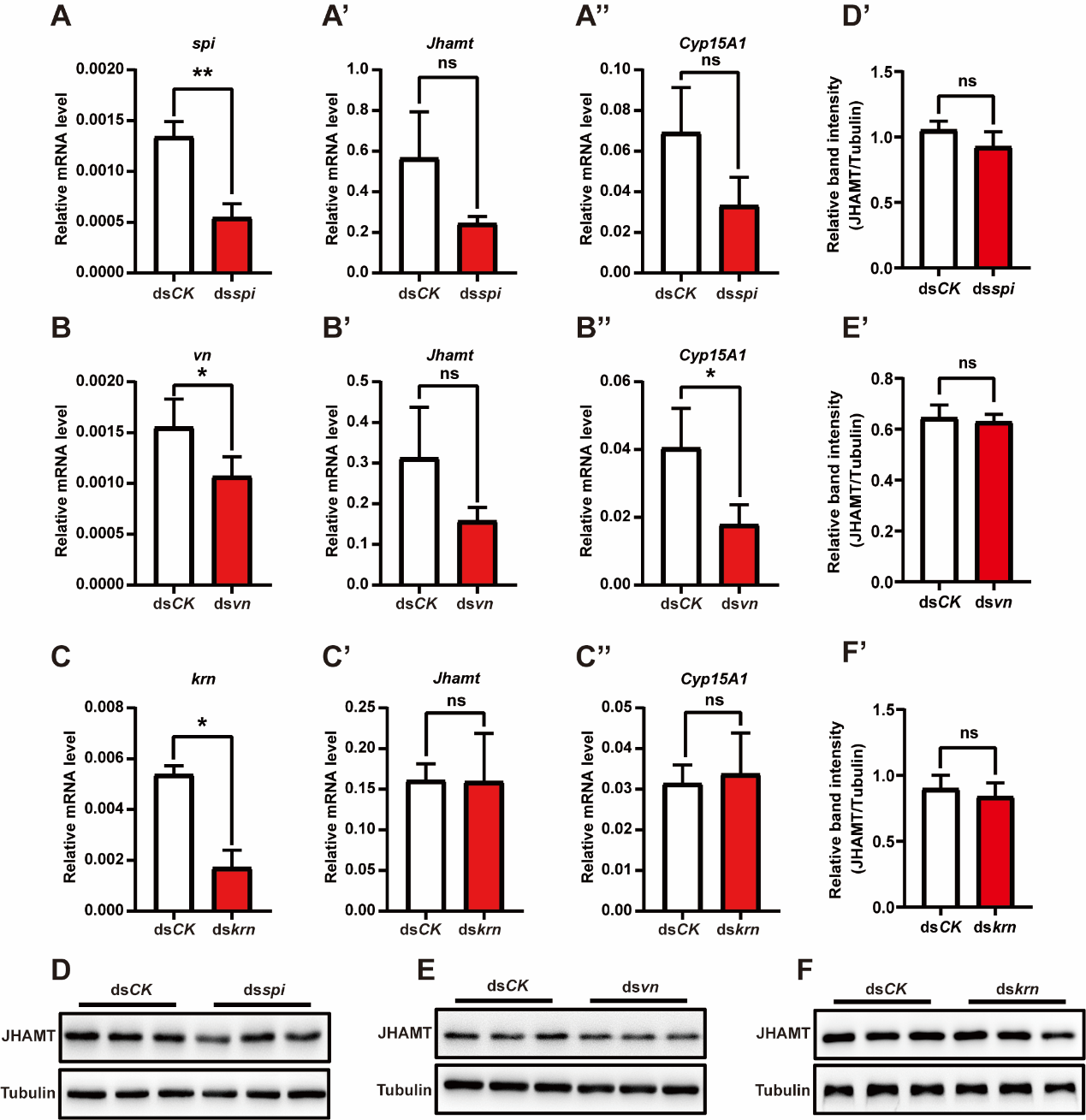


**Figure S1.** **Effect of RNAi each Egf ligand gene on *Jhamt* and *Cyp15A1* expression.** (A-A’’) Effect of *spi* RNAi on *Jhamt* and *Cyp15A1* expression in the head. (B-B’’) Effect of *vn* RNAi on *Jhamt* and *Cyp15A1* expression in the head. (C-C’’) Effect of *krn* RNAi on *Jhamt* and *Cyp15A1* expression in the head. (D-F) Western blotting analysis of JHAMT protein level. (D’-F’) Quantification of relative band intensity of JHAMT.


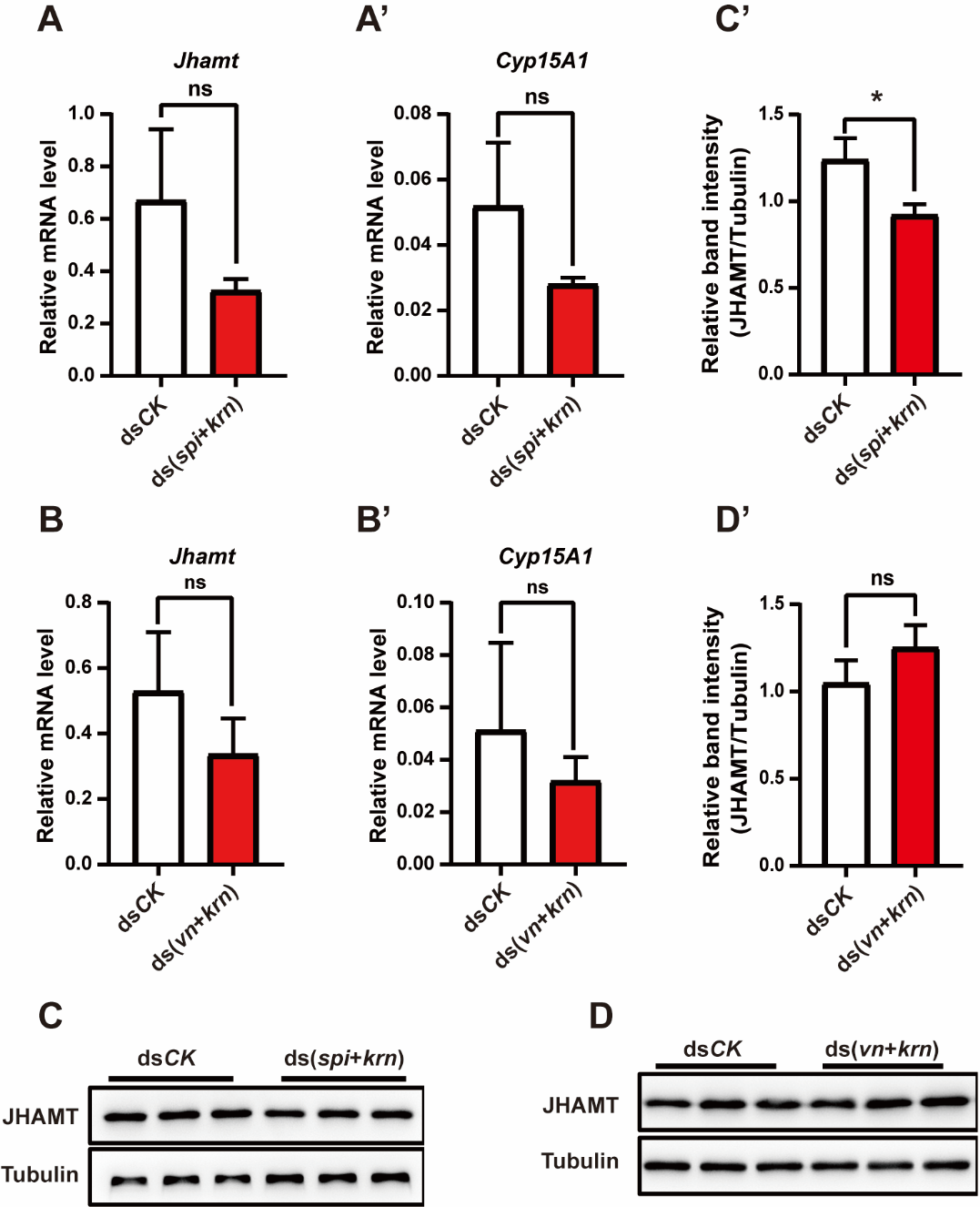


**Figure S2. Effect of RNAi pairwise combination of the three Egf ligand genes on *Jhamt* and *Cyp15A* expression.** (A-A’) Effect of simultaneous RNAi *spi* and *krn* on *Jhamt* and *Cyp15A1* expression in the head. (B-B’) Effect of simultaneous RNAi of *vn* and *krn* on *Jhamt* and *Cyp15A1* expression in the head. (C-D) Western blotting analysis of JHAMT protein level. (C’-D’) Quantification of relative band intensity of JHAMT.


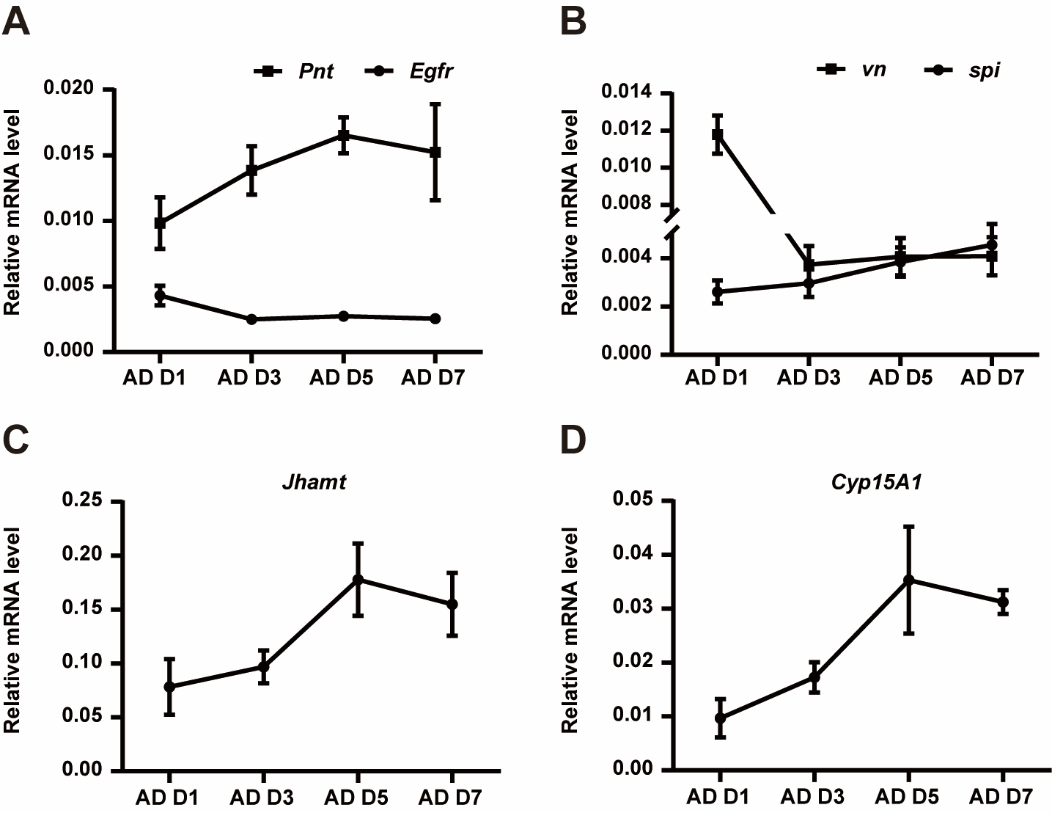


**Figure S3.** **Expression patterns of *Egfr*, *spi*, *vn*, *Jhamt* and *Cyp15A1* of** **adult females, during the first vitellogenic cycle.** (A) Expression patterns of *Egfr* and *Pnt* in the CA of adult females. (B-D) Expression patterns of *vn*, *spi*, *Jhamt* and *Cyp15A1* in the head of adult females.


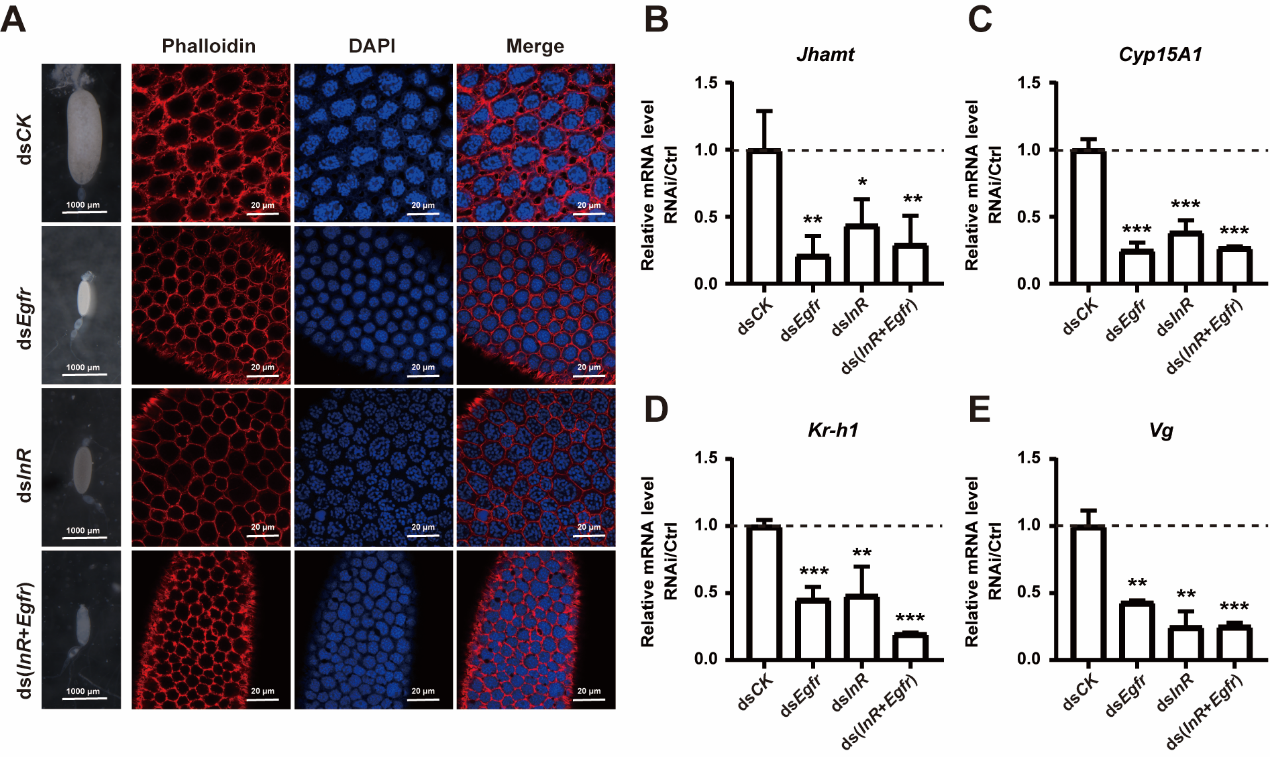


**Figure S4. Compare *Egfr* RNAi with *InR* RNAi.** (A) Ovarian phenotypes after RNAi *Egfr* and *InR*, and simultaneous RNAi of *Egfr* and *InR*. (B-C) Effect of RNAi *Egfr* and *InR*, and simultaneous RNAi of *Egfr* and *InR* on *Jhamt* and *Cyp15A1* expression in the head. (D-E) Effect of RNAi *Egfr* and *InR*, and simultaneous RNAi of *Egfr* and *InR* on *Kr-h1* and *Vg* expression in the fat body.


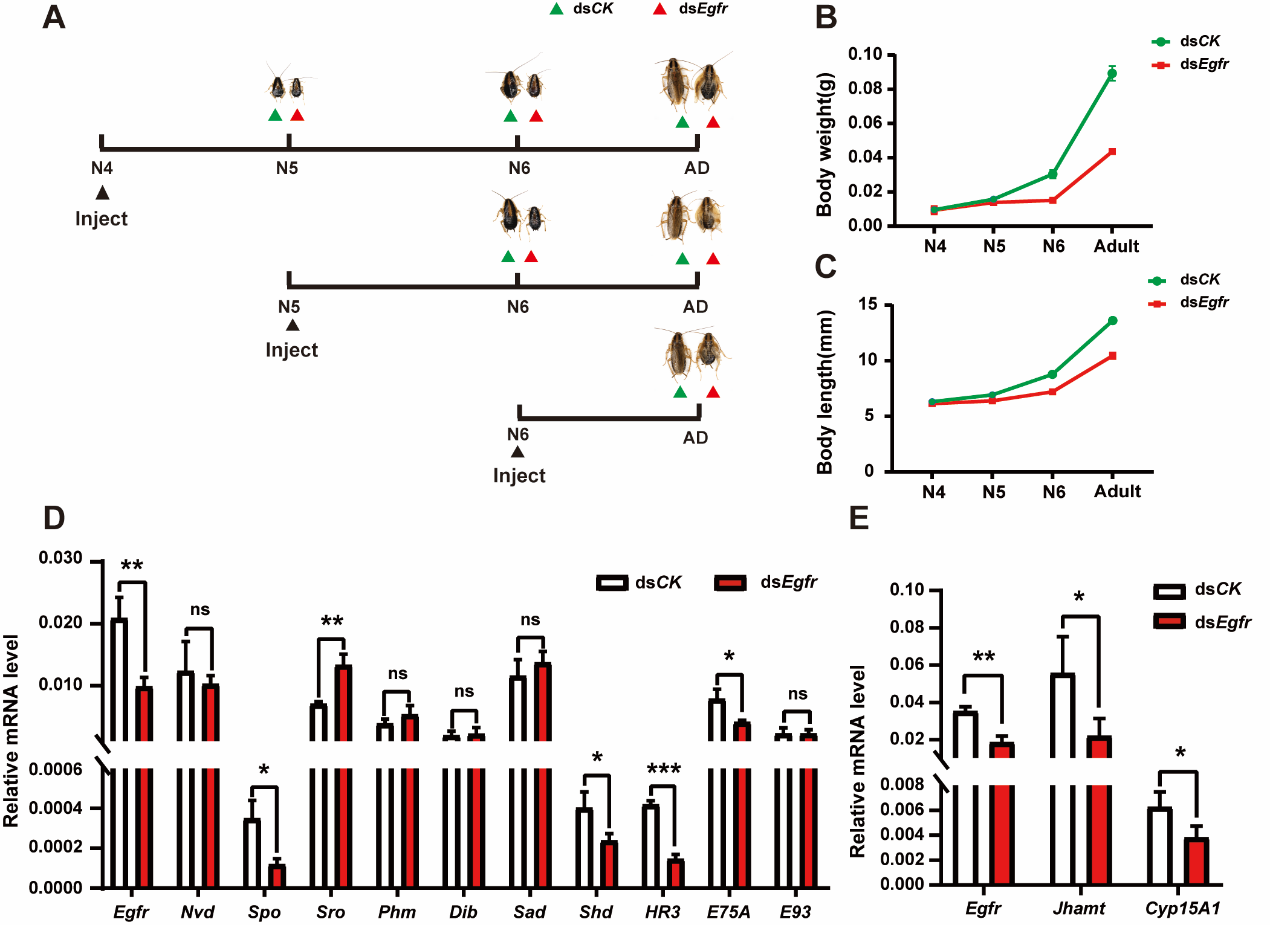


**Figure S5.** **RNAi *Egfr* at the nymph stage.** (A) The phenotype of *Egfr* RNAi at the nymph stage. (B-C) Effect of *Egfr* RNAi on the body size. The green line is the control animals and the red line is the *Egfr* RNAi. (D) Effects of *Egfr* RNAi on 20E biosynthesis and downstream primary response genes at N6. (E) Effects of *Egfr* RNAi on *Jhamt* and *Cyp15A1* expression at N5. AD, adult; N, nymph.


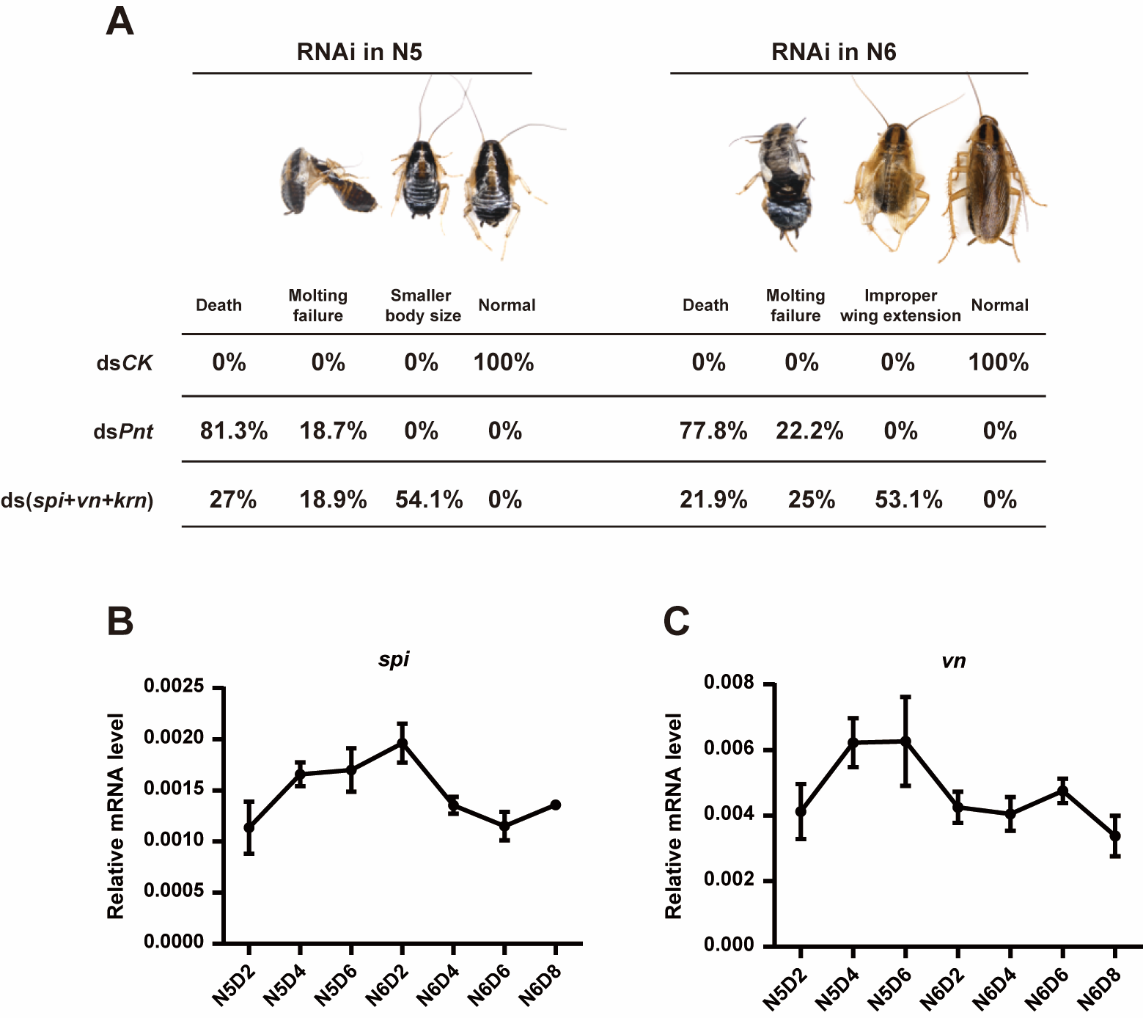


**Figure S6. Egf ligands and *Pnt* RNAi at the nymph stage.** (A) Phenotypes and statistics of RNAi Egf ligands and *Pnt* at N5 and N6. (B) Expression patterns of *spi* in the head, during the N5-N6. (C) Expression patterns of *vn* in the head, during the N5-N6. N, nymph.


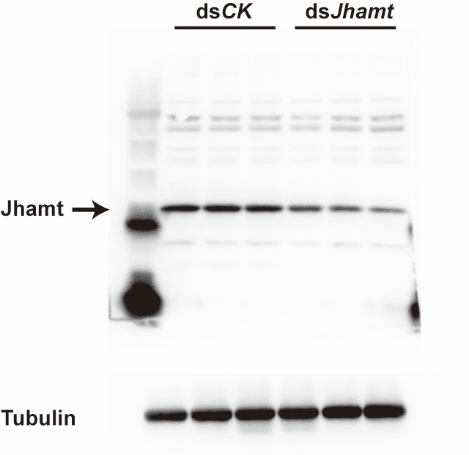


**Figure S7. Specificity verification of anti-JHAMT.** JHAMT protein levels in the head after *Jhamt* RNAi.

**Table S1 List of primers used in this study**

| **Name** | **Nucleotide sequence** |
| --- | --- |
| **dsRNA synthesis** | **5’-3’** |
| dsCK-Forward | TAATACGACTCACTATAGGGAAAGCTC |
| ds*CK*-Reverse | TAATACGACTCACTATAGGGAATACAGCGGCCGCGAG |
| ds*Egfr*-Forward | TAATACGACTCACTATAGGACTTGGAGATAATAGGAGGC |
| ds*Egfr*-Reverse | TAATACGACTCACTATAGGACCGTTGATGATCGCTTT |
| ds*Vein*-Forward | TAATACGACTCACTATAGGTTACCAACCAAGCCCTCT |
| ds*Vein*-Reverse | TAATACGACTCACTATAGGCACAATGTACTTCCCTCCAC |
| ds*Keren*-Forward | TAATACGACTCACTATAGGTGTAGCCGCAGACCGTATG |
| ds*Keren*-Reverse | TAATACGACTCACTATAGGGTTGGTATTGAACCAGGTGAGT |
| ds*Spitz*-Forward | TAATACGACTCACTATAGGGCCCCAACATCACATTCC |
| ds*Spitz*-Reverse | TAATACGACTCACTATAGGACTGGCTCGGGTTTCTTT |
| ds*Ras85D*-Forward | TAATACGACTCACTATAGGGCTGTTAATAGTGCGAAGTCCT |
| ds*Ras85D*-Reverse | TAATACGACTCACTATAGGGCGTCATCCACACCCATTC |
| ds*Pnt*-Forward | TAATACGACTCACTATAGGTACAGTGACGCCTACAGTTC |
| ds*Pnt*-Reverse | TAATACGACTCACTATAGGTCATTTTGGGTTTGTTCTT |
| ds*InR*-Forward | TAATACGACTCACTATAGGCCTGGAGTGAATGTGGATAG |
| ds*InR*-Reverse | TAATACGACTCACTATAGGTAACAGTAGCTCTTAGCTTTGTA |
| ds*Pvr*-Forward | TAATACGACTCACTATAGGTGGATGAGCAAGCGGAACT |
| ds*Pvr*-Reverse | TAATACGACTCACTATAGGACGAAGTGATCTCTGTGTCTGA |
| ds*Ddr*-Forward | TAATACGACTCACTATAGGCGATGGTGGTGCTGCTCTT |
| ds*Ddr*-Reverse | TAATACGACTCACTATAGGGGAGGCGTCAAGTTGTAGAATG |
| ds*Ror*-Forward | TAATACGACTCACTATAGGCCAGCACTGACACAACCTCATA |
| ds*Ror*-Reverse | TAATACGACTCACTATAGGACAATACCAGCACCACTTCCAA |
| ds*Alk*-Forward | TAATACGACTCACTATAGGGGTCACTTCCTGCTGTTACG |
| ds*Alk*-Reverse | TAATACGACTCACTATAGGTGTTGCTCATCTTCGCCATC |
| ds*Dnt*-Forward | TAATACGACTCACTATAGGTCATCGCCGTTATCGTCATCA |
| ds*Dnt*-Reverse | TAATACGACTCACTATAGGGCCACACCTTCAGCACTCA |
| ds*Tor*-Forward | TAATACGACTCACTATAGGTGCCGAGGACAGTGCTATC |
| ds*Tor*-Reverse | TAATACGACTCACTATAGGACCACCAAGTGTAACAATCTCC |
| ds*Eph*-Forward | TAATACGACTCACTATAGGGCGACATGACGATGGTAATGC |
| ds*Eph*-Reverse | TAATACGACTCACTATAGGTGCGGTGAACAGAGGAGTTG |
| ds*RYK*-Forward | TAATACGACTCACTATAGGACAGCCTCTACTCGTCTATCCT |
| ds*RYK*-Reverse | TAATACGACTCACTATAGGGTGCCAAGCGGTATCCATCT |
| ds*Cad96ca_0*-Forward | TAATACGACTCACTATAGGGTGCCGAGCGTTACTATGGA |
| ds*Cad96ca_0*-Reverse | TAATACGACTCACTATAGGGGACTTGACCGAGAAGATGTTG |
| ds*Fgfr*-Forward | TAATACGACTCACTATAGGCCTTCGTGAACTACAGCAAGA |
| ds*Fgfr*-Reverse | TAATACGACTCACTATAGGGCTTCTGTTTGACTTCCTTTGT |
| ds*Cad96ca_1*-Forward | TAATACGACTCACTATAGGGTGTGGCAAGAGGAATGGAGTA |
| ds*Cad96ca_1*-Reverse | TAATACGACTCACTATAGGCATTGTTGGATACGGCGTTGAT |
| ds*Nrk*-Forward | TAATACGACTCACTATAGGTGAATCCGAAGGTGCGATGG |
| ds*Nrk*-Reverse | TAATACGACTCACTATAGGTGTGCTCCTGTCCGATGGTA |
| ds*Ret*-Forward | TAATACGACTCACTATAGGCCAGGAGTTGCTGTTCACAATC |
| ds*Ret*-Reverse | TAATACGACTCACTATAGGCGCTAGTAGTCGCTGTTCATCT |
| ds*Otk*-Forward | TAATACGACTCACTATAGGGACGGTGGTGATGGTGAAGT |
| ds*Otk*-Reverse | TAATACGACTCACTATAGGCGGTAAGGTTGGAAGCAATGAG |
| **qPCR** |  |
| *Actin5C*-Forward | TGAGACCACATACAACTCCA |
| *Actin5C*-Reverse | CAATTCCAGGGTACATGGTG |
| *InR*-Forward | TGTATGATGGACGAGACGCA |
| *InR*-Reverse | CAGTCCTCTCGGTTGCAATG |
| *Pvr*-Forward | CCAAGTGGAGAGCCAGATGA |
| *Pvr*-Reverse | CAGCCAGATCACCATGCAAT |
| *Ddr*-Forward | TGGTGGGTGAAGAATCGGAA |
| *Ddr*-Reverse | CTCCGCTCTTGCTTGAAGAC |
| *Ror*-Forward | CAATGGCGGTCAAGCTGTAA |
| *Ror*-Reverse | GCTGCTGCCTAACATTTGGT |
| *Alk*-Forward | ACTCCGCTGTCATGGAAGAA |
| *Alk*-Reverse | CCTTGTCCAATGCTGGGATG |
| *Dnt*-Forward | GTGTATTGCTGTGGGAGCTG |
| *Dnt*-Reverse | TAGGTTGAGCAAGGCGGTAA |
| *Tor*-Forward | CACAGAAGACCTCCGTCAGT |
| *Tor*-Reverse | GCAGCCTATGAGCGAAACAA |
| *Eph*-Forward | CAATGATGGCACAGATTCCGA |
| *Eph*-Reverse | CAACCGTGGGTTGTCTTAGC |
| *RYK*-Forward | AACTGTGTGGTGGACGAGAA |
| *RYK*-Reverse | TGCAAGCCACTTGATTGGTC |
| *Cad96ca_0*-Forward | GCAGATCCGCTGTTTGTGAT |
| *Cad96ca_0*-Reverse | ACGCGAATTCCTCAAGAAGC |
| *Fgfr*-Forward | CACCTTCGTGAACTACAGCA |
| *Fgfr*-Reverse | CTTGCTGACGGTATTTGGACA |
| *Cad96ca_1*-Forward | GCATCACCACCATCATCACC |
| *Cad96ca_1*-Reverse | ATCACAGGCAATGCGACTTC |
| *Nrk*-Forward | GCCAACATTGGGACTCTCAG |
| *Nrk*-Reverse | CGCACCTTCGGATTCATTGT |
| *Ret*-Forward | AGCTTTGGTGTTCTCATGTGG |
| *Ret*-Reverse | TGAACAGCAACTCCTGGGTA |
| *Otk*-Forward | CATAGAGAAGGCGCTGGAGA |
| *Otk*-Reverse | CTCTGCGTAGAGCTGTGAGA |
| *Egfr-*Forward | CCAGGTCCTGACCAATGTCT |
| *Egfr*-Reverse | GCTAACAGGGCACACTGAAG |
| *Kr-h1*-Forward | GCGAGTATTGCAGCAAATCA |
| *Kr-h1*-Reverse | GGGACGTTCTTTCGTATGGA |
| *Vein*-Forward | CTTCCAATATCGCCGCCTTC |
| *Vein*-Reverse | ACATTGTACACACCGTCCCT |
| *Keren-*Forward | TGTAGCCGCAGACCGTATG |
| *Keren-*Reverse | GTTGGTATTGAACCAGGTGAGT |
| *Spitz-*Forward | TAGCAGTGTTCCTGGTCGTT |
| *Spitz*-Reverse | GCTGAACGGTCGTCTTTCTC |
| *Argos-*Forward | TGTTAGAAAGCGCCAAGAGC |
| *Argos-*Reverse | CCGTGAGGACACTGGCATAA |
| *Ras85D*-Forward | TCTTCTCGATATCCTGGACACA |
| *Ras85D-*Reverse | GACTTCGCACTATTAACAGCAA |
| *Pnt*-Forward | GCAAGCGTTATGTGTACCGT |
| *Pnt-*Reverse | TCTGGCTTCAAGTCCACCAT |
| *Jhamt*-Forward | GACCTGGTGGTGAAGTCTTGG |
| *Jhamt-*Reverse | TGACTCCATTTCGATTTTTTACTCTG |
| *Cyp15A1*-Forward | ATGATGCGTTTGACGTGTGT |
| *Cyp15A1-*Reverse | GGAGACGCTGATCATCCAAT |
| *Met*-Forward | GACAGATGTATGATCGTGGC |
| *Met-*Reverse | AGTGTCCTTGTCGTATTCCA |
| *Nvd*-Forward | CTGGGGCCAGTCACAATACT |
| *Nvd-*Reverse | GCAGGGGCTTGTCAATGTAT |
| *Phm*-Forward | CTAGGCACCAGAGCACCTTC |
| *Phm-*Reverse | GCAAGCACTGTGTCTTCCAA |
| *Dib*-Forward | ACCAGATCCTGAGTCTCCAA |
| *Dib-*Reverse | TAGACGGCAAGCTACTTGGT |
| *Sad*-Forward | ATGAGGAGGTTCAGGGTGTG |
| *Sad-*Reverse | CTGGCCAGAAGTCATTTGGT |
| *E75*-Forward | GTGCTATTGAGTGTGCGACATGAT |
| *E75-*Reverse | TCATGATCCCTGGAGTGGTAGAT |
| *Hr3*-Forward | GATGAGCTGCTCTTAAAGGCGAT |
| *Hr3-*Reverse | AGGTGACCGAACTCCACATCTC |
| *Sro*-Forward | AGCAGTCATTGTGCGTTAGC |
| *Sro-*Reverse | TGAGTCCATCACACCAAGCA |
| *Shd*-Forward | AACACATTCCAGGACCTCGT |
| *Shd-*Reverse | TGTAAGCATCGTGCACTTGG |
| 5’-Race |  |
| 5’-Race- Cyp15A1_Reverse 1 | CCACATTGGGCCCTCCGTGAATAC |
| 5’-Race- Cyp15A1_Reverse 2 | CACACGTCAAACGCATCAT |
