# Supplementary figures and images for: Egfr signaling promotes juvenile hormone biosynthesis in the German cockroach
Source: BMC Biol. 2022 Dec 13;20:278. doi: 10.1186/s12915-022-01484-z (PMC9749228; doi:10.1186/s12915-022-01484-z)

**Figure 2D**

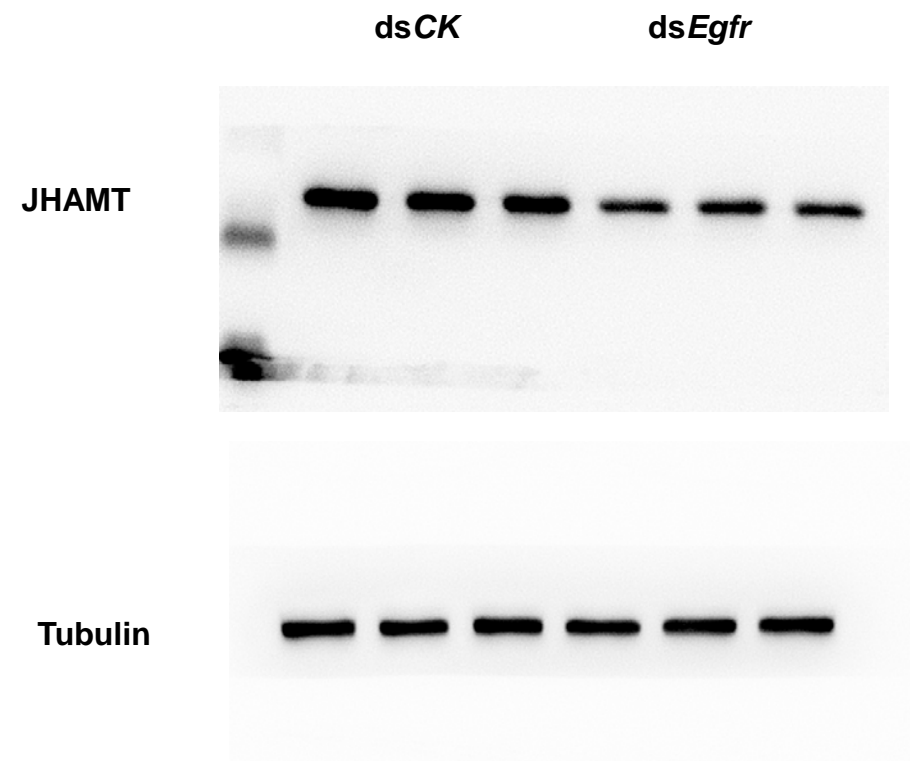

Figure 3D

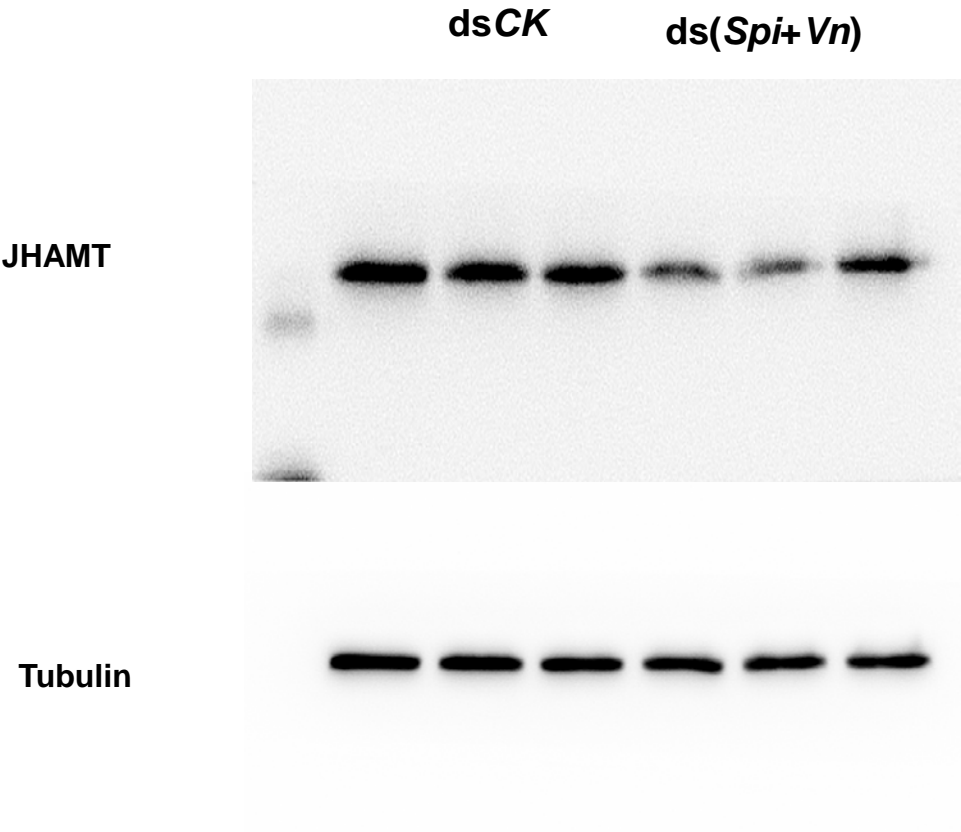

Figure 3E

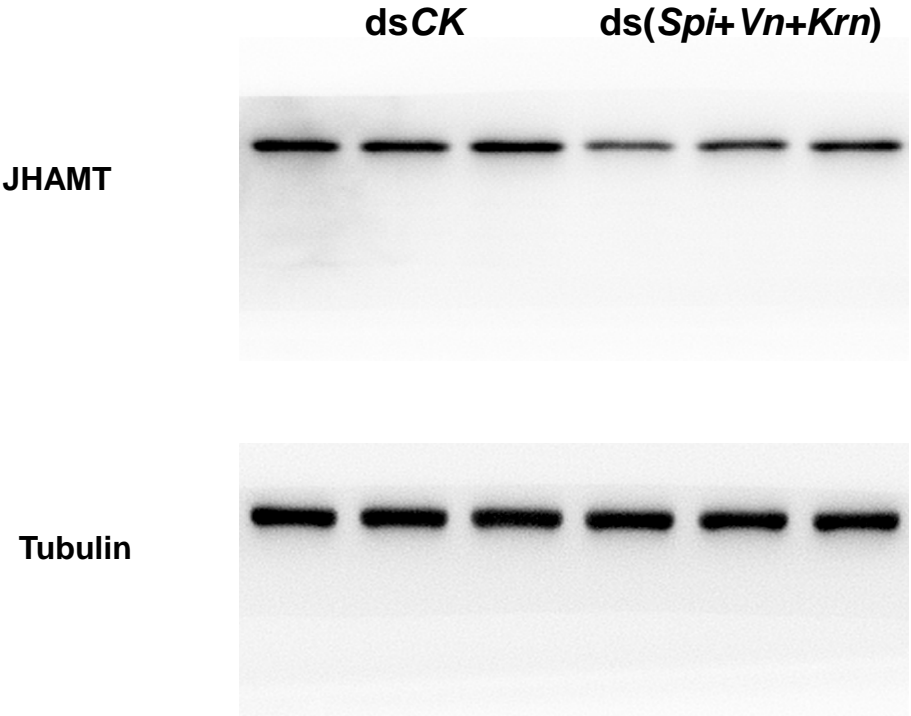

Figure 4A

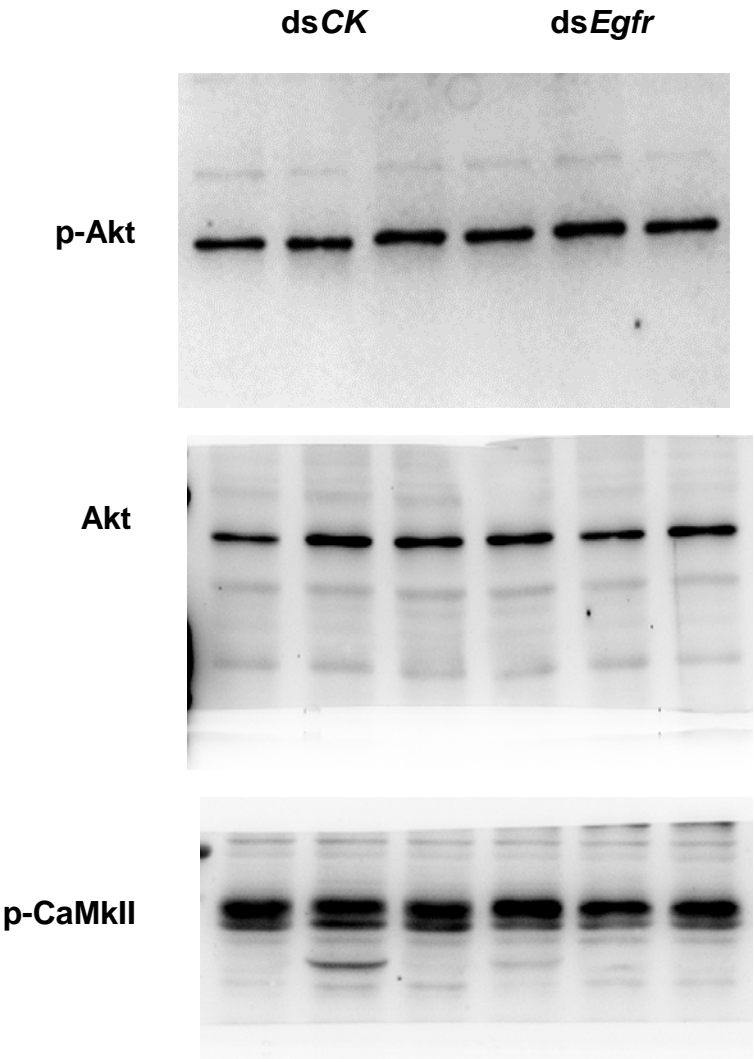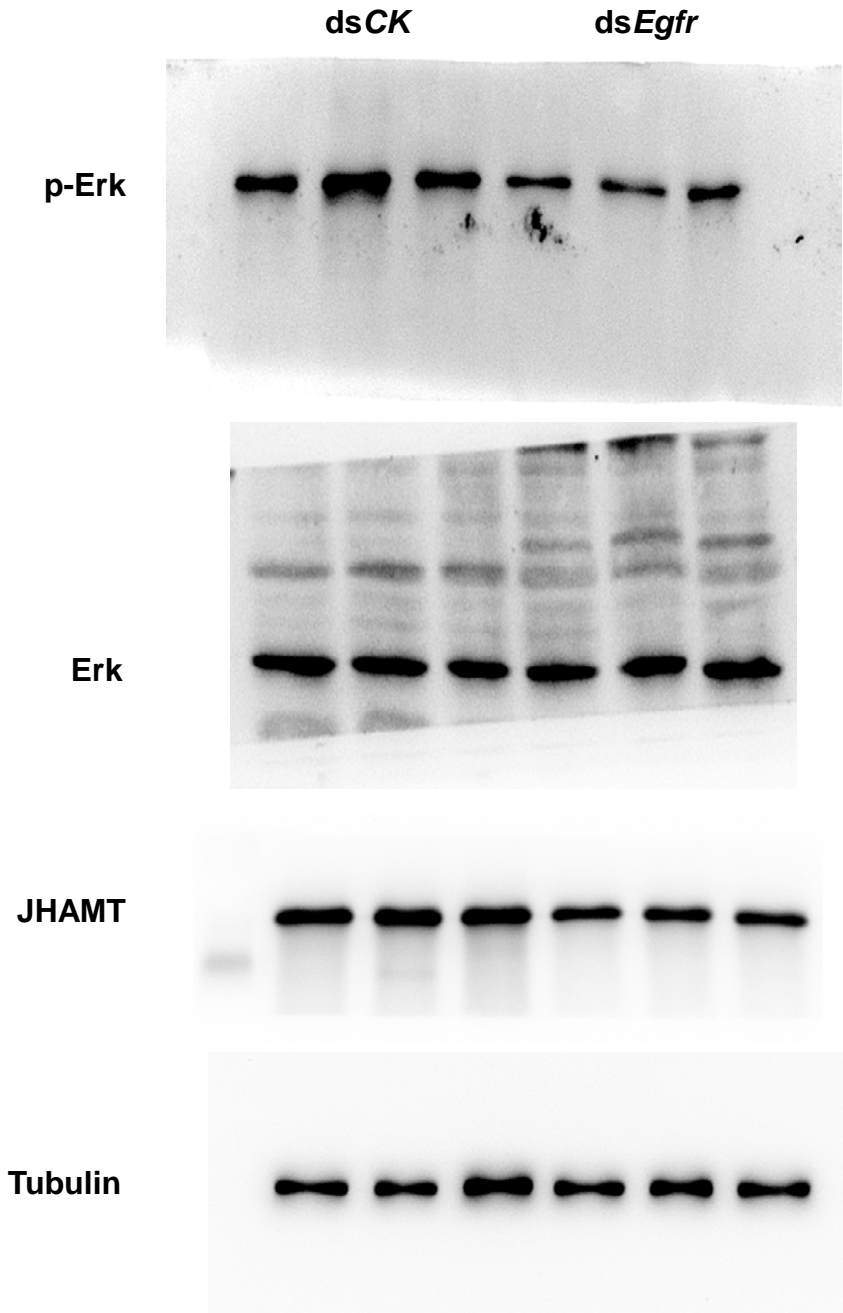

Figure 4B

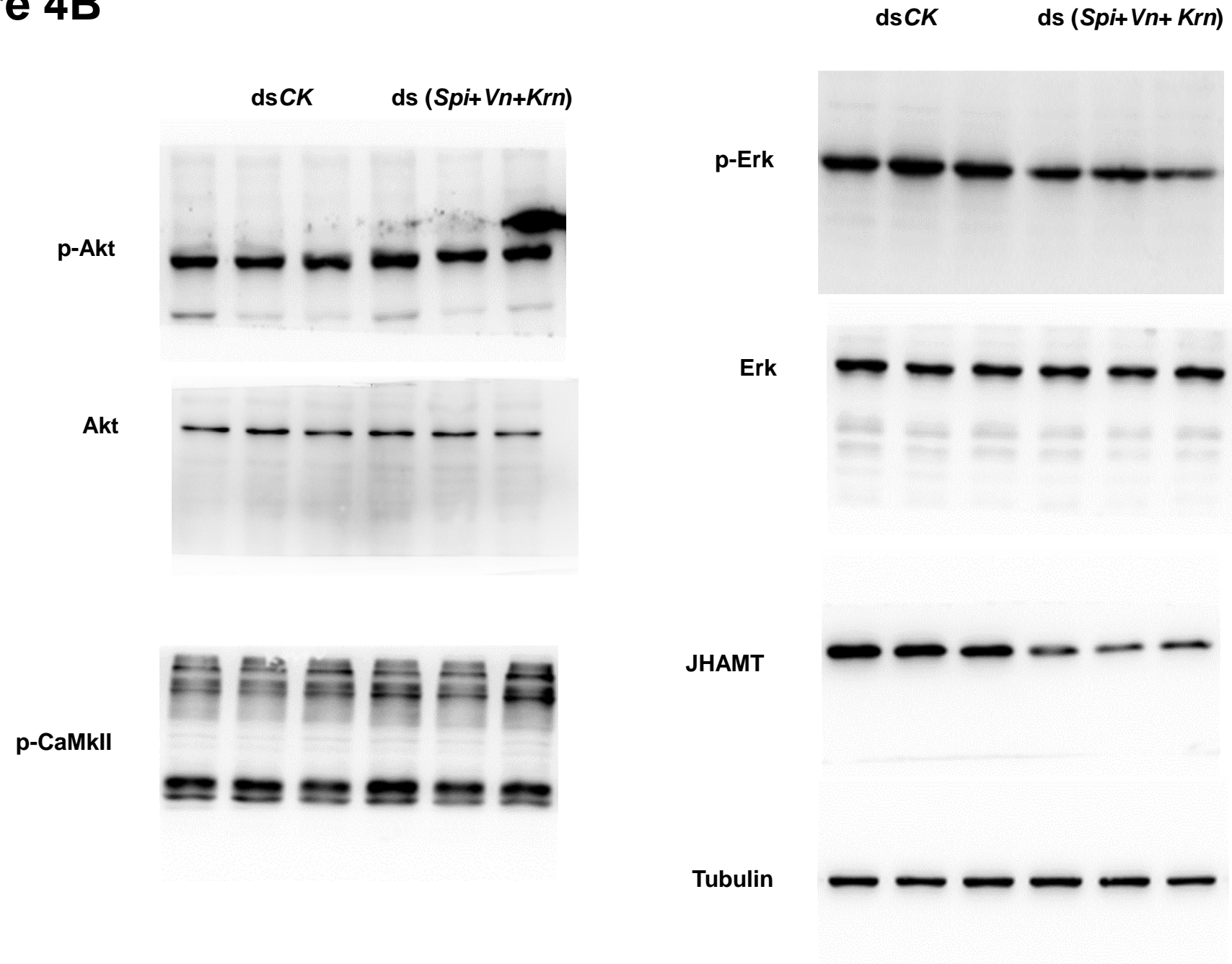

**Figure 4F**

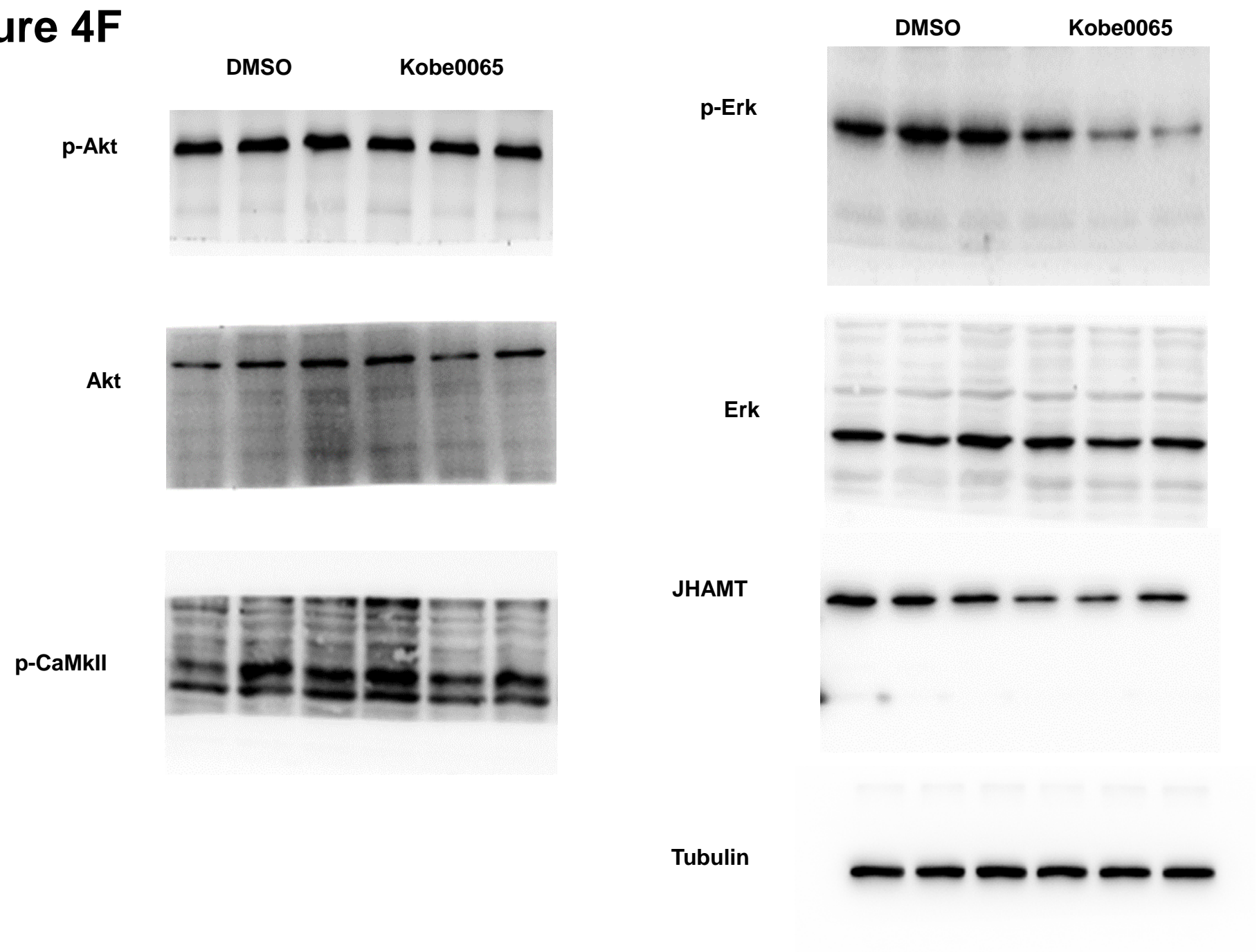

Figure 5C

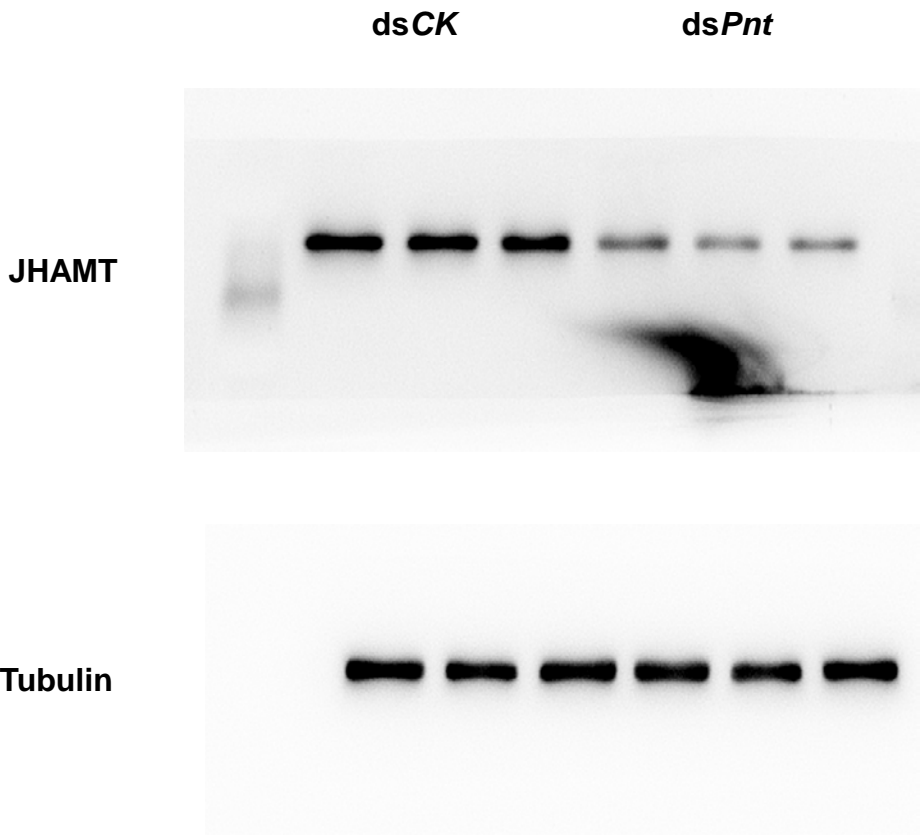

Figure S1

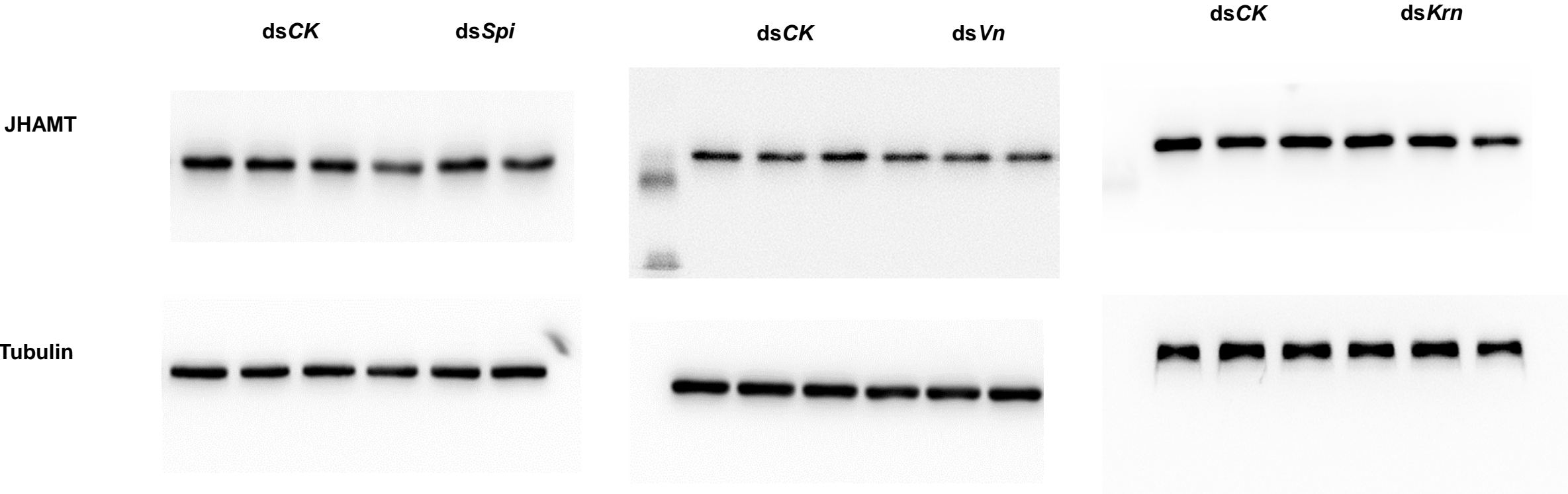

**Figure S2**

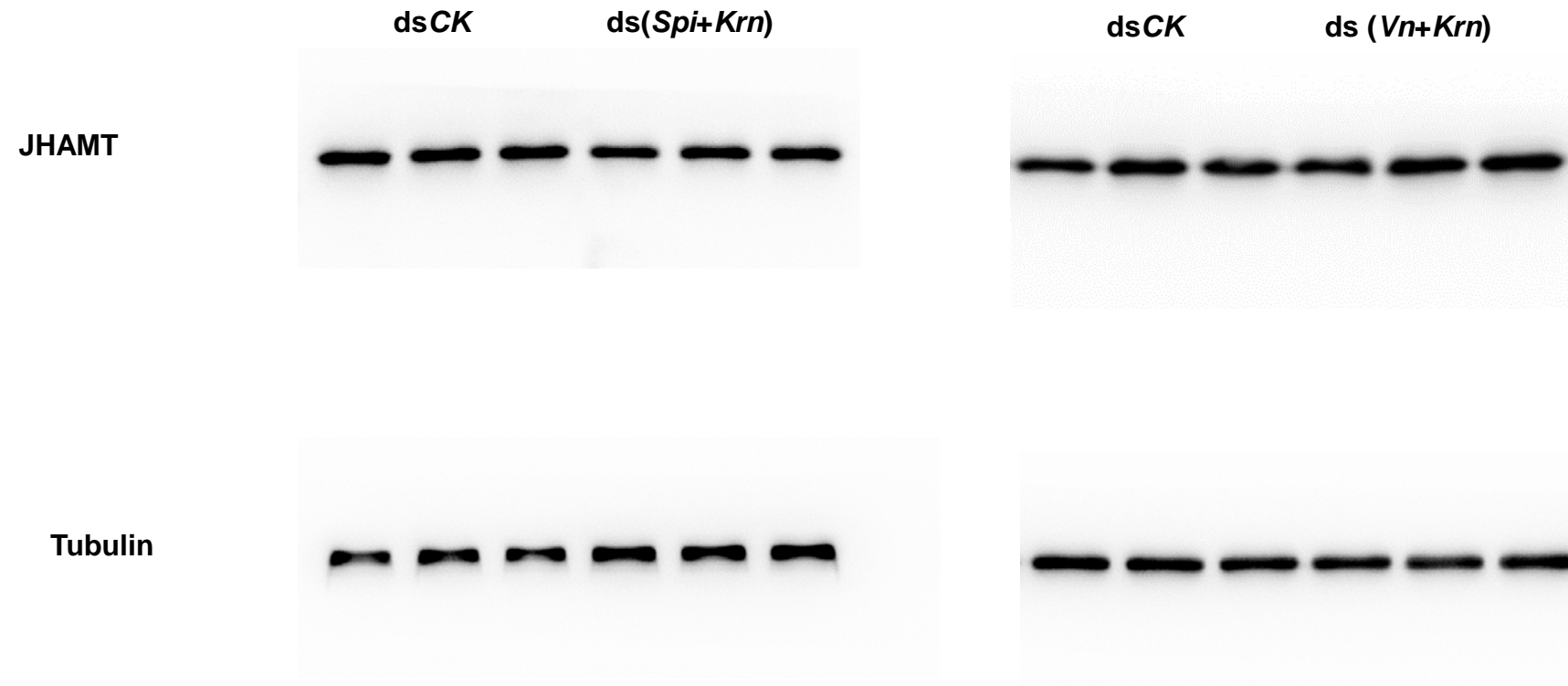

**Figure S7**

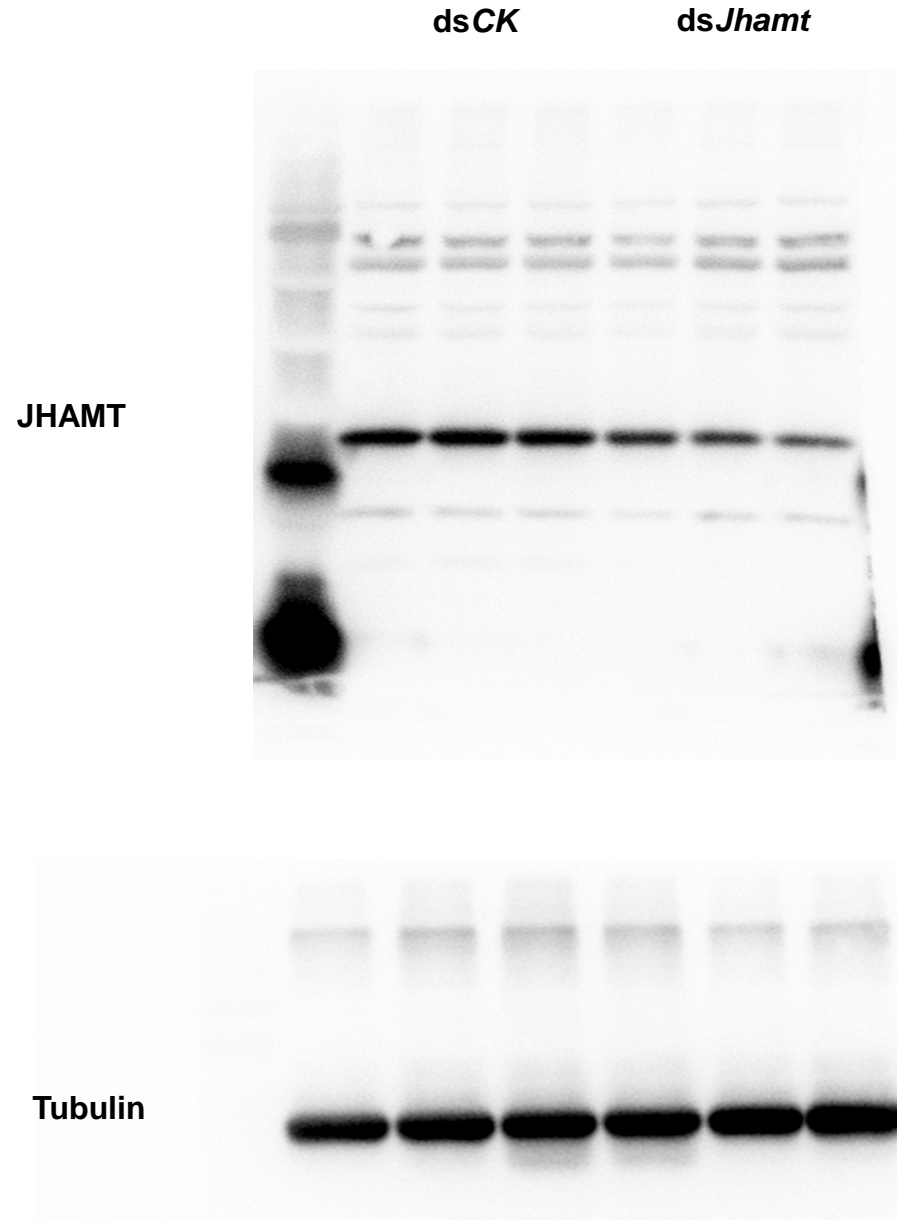

Supplement: Supplementary file 3 — Additional file 3. Original Western blot data. [file 12915_2022_1484_MOESM3_ESM.pdf]
